# The Role and Future of Functional Graphenic Materials in Biomedical and Human Health Applications

**DOI:** 10.1021/acs.biomac.4c01431

**Published:** 2025-03-18

**Authors:** Anne M. Arnold, Juhi Singh, Stefanie A. Sydlik

**Affiliations:** † Department of Chemistry, Carnegie Mellon University, 4400 Fifth Avenue, Pittsburgh, Pennsylvania 15213, United States; ‡ Department of Biomedical Engineering, Carnegie Mellon University, 346 Hamerschlag Drive, Pittsburgh, Pennsylvania 15213, United States

## Abstract

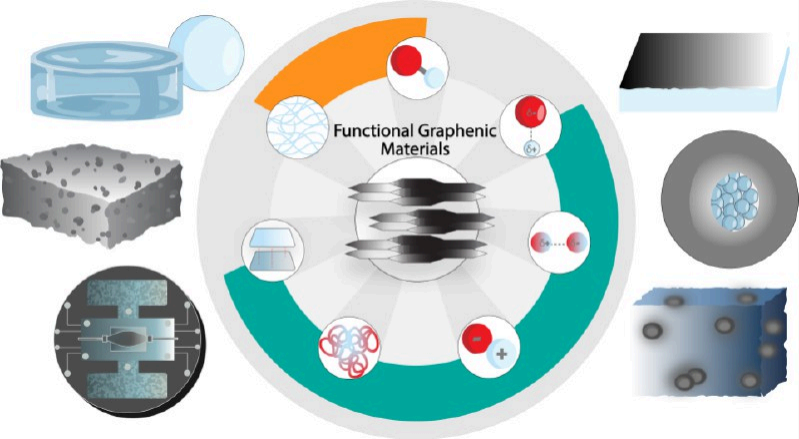

Functional graphenic materials (FGMs) are materials derived from graphene oxide (GO) that hold a plethora of applications from electronics to nanomedicine. In this Perspective, we examine the history and evolution of biomedical applications of this carbon-based macromolecule. Following the carbon nanotube (CNT) movement, GO and FGMs became nanocarbons of interest because of their low cost and versatile functionality. The tunable chemistry enabled our work on FGMs coupled with biomacromolecules and allows FGMs to plays an important role in many biomedical applications, from tissue regeneration to controlled delivery. As we work to develop this material, it is critical to consider toxicity implications—in fresh materials as well as in degradation products. With this understanding, FGMs also hold potential roles in human health and environmental sustainability, making FGMs an important contemporary biomacromolecule.

## 1 History of FMGs and Classic Applications

A first clarification must be made in the classification of functional graphenic materials (FGMs) and graphene oxide (GO). Certainly, with a carbon backbone, FGMs are organic. With the plethora of oxygen-containing functionalities that decorate the carbon backbone, GO is a veritable playground for an organic materials chemist. However, most materials chemists who work with heterogeneous materials are inorganic chemists, while most organic materials chemists concern themselves with polymers. Since the chemistry of GO bears more in common with polymers than inorganic materials such as quantum dots, GO can be classified as a macromolecule. Indeed, most experts in the FGM space, including greats like Swager and Tour, are undoubtedly polymer chemists. Thus, this examination of the future of FGMs as a biomacromolecule fits within the scope of this journal.

### 1.1 Graphene Oxide

GO became recognized as a material for research through the publication of its seminal synthesis by Hummers in 1958.^1^ The synthesis was classically developed and seen as a route to reduced graphene. The Hummers method led to highly exfoliated flakes of GO that could then be reduced via chemical or thermal means. This popular synthetic method has been heavily used and is accepted as the “gold standard” method for GO synthesis. However, there are many drawbacks to this method, including uneven flake size, heterogeneous distribution of layers, and destruction of the conjugated π system, which is responsible for the superior mechanical and electronic properties that are sought in graphene-based materials. Because most work through the early 2000s focused on the development of graphene-based materials for mechanical and electronic properties,^2−5^ there are numerous reports of improved or modified Hummers methods.^6,7^

**Figure 1:**
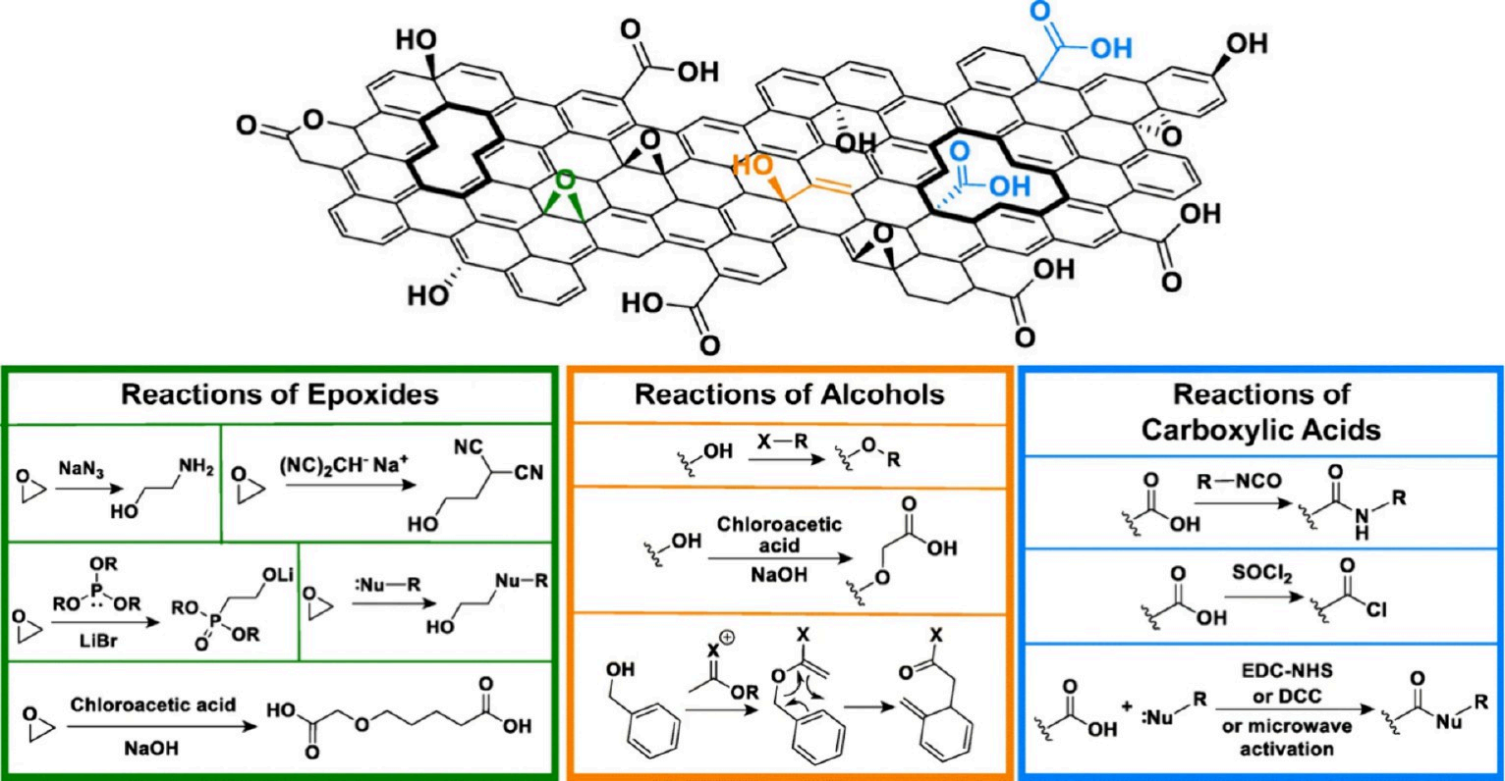
Traditional organic chemistry reactions can be employed to covalently functionalize the alcohols, epoxides, and carboxylic groups present on the edges, on the basal plane, and around defects of a sheet of GO. Reproduced with permission from ref (24). Copyright 2018 The Regenerative Engineering Society.

GO is an oxidized analogue of graphene, consisting of a disrupted π-conjugated basal plane of carbons, decorated with oxygen functionalities including alcohols, epoxides, and carboxylic acids (Figure 1). The early 2010s were rife with research focused on improving the synthesis of GO,^3^ and with improved synthetic control, came a better understanding of the chemical landscape. Carbon-to-oxygen ratios lower than 1:1^2^ confirm the presence of adsorbed water, which remains even after extensive vacuum drying and can greatly affect the chemistry. The density, placement, and type of oxygen functionalities can be controlled, to some degree, through the conditions used for synthesis.^8^ For example, the epoxide can be favored with benzoyl peroxide,^9^ or carboxylic acids can be favored over alcohols by logically adding additional equivalents of potassium permanganate during synthesis.^10^ The increased carboxylic acid content is likely due to the potassium permanganate introducing more defects in the sheets, as carboxylic acids are often found at defect sites and along sheet edges. Additionally, we have observed that higher amounts of potassium permanganate led to fewer epoxides and more alcohols, likely due to the degradation of epoxides into alcohols under the harsh oxidizing conditions. The methods for quenching and workup can also alter the functional group distribution. While many methods for “green” synthesis of GO have been reported,^6^ and many modifications made,^7^ Hummers’ method for GO synthesis remains the gold standard in the field for preparation. Its efficiency, high yield, consistent quality, scalability, and adaptability make it the preferred approach for both research and industrial applications.

### 1.2 Reduced GO

The reduction of GO was also important in the early 2010s and critical for the future applications in biomedicine. Reduced GO (rGO) rose in popularity because it is easier to access and handle than graphene and offers some restoration of the mechanical and electrical properties lost in the oxidation to GO. Hydrazine was one of the first reagents employed for this purpose; however, as rocket fuel, the reaction had to be handled with care. Sodium borohydride (NaBH_4_) was a next step, offering much gentler, but still reactive conditions. The classic reactive counterpart to NaBH_4_, lithium aluminum hydride (LiAlH_4_), provides an interesting reaction. Rather than reducing the basal plane of graphene, LiAlH_4_ provides functional group reduction of the carboxylic acids on sheet edges and defect sites to alcohols.^4^ A popular approach for the gentle reduction of GO was the use of vitamin C, which offered significant reduction, with limited synthetic hazards.^11^ While thermal reduction appears to be a straightforward approach, many who have attempted it have encountered this unpredictable explosive reaction,^5^ termed autoignition. This reaction occurs unexpectedly during heating, resulting in the transformation of reduced graphene oxide (rGO) into an entirely insoluble and unpredictable black material. Because of this explosive potential, all reactions with GO should be undertaken with care and behind a blast shield. This is especially important for brave researchers who want to access GO or rGO through ball milling^6^ or microwave synthesis. Since this time, we have found that the level of oxidation of GO can be critical for biocompatibility, with rGO offering better compatibility^7^ for reasons we will later discuss.

Following in the footsteps of carbon nanotube (CNT) functionalization popularized in the 2000s, researchers of the early 2010s were eager to create functional graphenic materials (FGMs). It was here that I began my work with GO and FGMs at MIT under the direction of Tim Swager. FGMs prepared from GO have largely focused on nucleophilic attack on carboxylic acids; however, many creative methods for GO functionalization, such as aziridine functionalization or epoxide opening were developed during the early 2010s as well. These modifications served as a critical platform for the functionalization of GO with biomacromolecules in the coming decade.

Before turning our attention to functional graphenic materials, it is important to note that GO itself is still far from a mature field of research. Several areas still haunt chemists in the synthesis and characterization of GO, which remain important areas of research into the 2020s and beyond. An ongoing challenge is understanding the abundance and spatial distribution of functional groups. Determining their quantity is particularly difficult due to the complexity of characterizing graphene oxide, which typically requires intricate peak deconvolution of X-ray photoelectron spectroscopy (XPS) and solid-state carbon nuclear magnetic resonance (NMR) spectroscopy spectra. This process demands considerable expertise, and despite best efforts, some functional group peaks cannot be resolved. With respect to the spatial distribution of functional groups, researchers only recently reported mapping using atomic force microscopy (AFM)^6,7^ or infrared spectroscopy (IR).^12,13^ Even in these recent reports, it still remains difficult to distinguish between various oxygen functional groups, despite the differences in reactivity between epoxides (electrophiles) and alcohols (nucleophiles). Additionally, synthetically creating GO with monodisperse flake size and layers remains a challenge, with many reports still relying on fractionation to achieve this aim.^14^

### 1.3 Functional Graphenic Materials

FGMs do not try to perfectly capture the properties of graphene, but rather utilize the concepts of molecular design from classic polymer chemistry to create functional materials. For the purposes of this Perspective, an FGM is a GO analogue that has been modified to incorporate a polymer or small molecule using covalent chemistry. FGMs provide an intersection between polymer chemistry and 2D materials, as many of the reaction principles from polymer chemistry—including long reaction times and low solubility—apply for the synthesis of FGMs. Additionally, many FGMs have been synthesized that put functional polymer chains on the surface of GO, creating fuzzy graphenic sheets with improved compatibility in polymer dispersions. Functionalization of GO relies on the plethora of oxygen functionalities along the carbon backbone, using organic reactions to install new moieties (Figure 1). Numerous reviews have been published that provide in-depth summaries of the chemistries involved in the creation of FGMs.^2,4,15^ However, most functionalization chemistries fall into three major categories: functionalization using (1) carboxylic acids, (2) epoxides and other electrophiles, and (3) alcohols and other nucleophiles.

#### 1.3.1 Carboxylic Acids

Carboxylic acids are the most common functional handle used in GO functionalization. This potent electrophile dominates the edges of the graphene sheets and since the edges are less sterically encumbered than the basal plane, requires little to encourage reaction. Commonly, functional groups are attached using an amine-based nucleophile. To ensure greater functionalization efficiency, the carboxylic acid can be activated, through transformation to an acid chloride or use of carbodiimide reagents. One of the most popular in this class of reaction is the use of 1-ethyl-3-(3-(dimethylamino)propyl)carbodiimide (EDC) and *N*-hydroxysuccinimide (NHS), as these reagents are water-soluble, the chemistry relatively simple, and the process somewhat biocompatible.

#### 1.3.2 Epoxides and Other Electrophiles

Epoxides are also a potent electrophile abundantly available, more so on the basal plane of graphene oxide. One challenge of using this reactive site is that many nucleophiles typically used for epoxide opening are highly basic. This high basicity is incompatible with the adsorbed water associated with GO as well as the numerous carboxylic acids and alcohols with acidic protons. However, nucleophiles have been selectively used to open these epoxides with great efficiency to form FGMs. For example, stabilized carbanions were used as water-compatible anionic nucleophiles to create new carbon–carbon bonds on the graphene basal plane.^16^ In our group, we found that functionalization using the Arbuzov reaction primarily favors attack by the phosphorus nucleophile on the epoxide electrophile.^17^ Another interesting functionalization reports the use of the epoxide in a tandem functionalization method, using a thiol to open the epoxide, and then capitalizing on the new nucleophile formed by the oxygen after opening to perform a Michael addition.^18^

#### 1.3.3 Alcohols and Other Nucleophiles

The use of alcohols on the basal plane of GO as nucleophiles is one of the trickier functionalization methods, due to steric considerations. The tertiary alcohols on the basal plane are quite hindered, and on edges, alcohols are outnumbered by carboxylic acids. Despite this, there have been reports of using this nucleophile, especially with activation.^2^

To circumvent steric issues, the extended π-system can be considered, and the alcohols visualized as vinyl alcohols. As vinyl alcohols, these functionalities can be successfully subjected to Claisen rearrangements. The first report of this type of functionalization used an Eschenmoser–Claisen reaction to install amides, which are easier to characterize and definitively quantify the extent of functionalization using the atomic nitrogen content.^19^ This report was followed by my development of the more useful Johnson Claisen rearrangement, which installs reduction-proof esters or carboxylic acids on the basal plane of graphene, placed one methylene spacer away from the basal plane.^20^ My group has used this chemistry extensively in the preparation of FGMs for biomedical applications.

#### 1.3.4 Other Chemistries

Beyond the use of the functional groups on GO, other functionalization chemistries were borrowed from the carbon nanotube arsenal and adapted for preparation of FGMs. For example, diazirine derivatives can be used as carbene precursors to covalently tether a group to the graphene basal plane using a cyclopropane ring.^21,22^ Similarly, functionalities terminated in azides can be prepared as precursors to nitrenes, which can be used in a [1 + 2] cycloaddition reaction. Using the right reagents, this chemistry can open the door to facile tandem functionalization.^23^

### 1.4 Classic Applications of FGMs (Prior to 2013)

During the early days, synthesis of FGMs and GO composites were mostly focused on applications that utilized the electronic, thermal, and mechanical properties of graphene. Functionalization and composites focused on using FGMs or rGOs that leveraged these properties. In the Swager lab, for example, we used a negatively charged reduced Claisen graphene FGM to create a nanocomposite with polypyrrole. This material showed promise as a supercapacitor, with a capacitance up to nearly 280 F/g,^25^ and other groups reported its use in ultracapacitors.^26^ Other groups leveraged the thermal properties of graphene oxide,^27^ while many reports during this time focused on electronic properties,^28^ with applications in polymer-hybrid solar cells^29^ and transistors.^30,31^ FGM and GO sensors that utilized conductivity or resistivity to create a low-cost, low-energy sensor were also extremely promising.^3,32^

#### 1.4.1 GO Degradation in Water: A Path to Biomedical Use

These early works did little to elucidate the potential of FGMs in the field of biomaterials. However, in 2013, one of my last publications in the Swager group reported the use of the Arbuzov reaction to install hydroxyapatite-like polyphosphate groups onto GO and excitingly, this FGM exhibited bone-like mechanical properties. Around the same time, the Tour group published a seminal work demonstrating that GO was not only biodegradable, but *auto* degradable in water.^33^ This work opened the door between FGMs and biomaterials, and 2013 saw a significant uptick in papers concerning the toxicity of GO and exploring its application in biomaterials. A Google Scholar search for “Graphene Oxide Biomaterial” between 2013 and 2023 shows a 629% increase in publications, compared to a 370% increase for papers using only “Graphene Oxide”. It is important to note that post-2020 publication numbers may be skewed lower due to the impact of the COVID-19 pandemic on research output. Scientists began to consider FGMs as biomaterials, and the first area of focus was verifying GO compatibility and nontoxicity.

## 2 GO Toxicity

The Tour group’s discovery in 2013 revealed that graphene oxide degrades into a humic acid-like material in water,^33^ a surprising result given the stability typically associated with carbon-based nanomaterials. The study demonstrated that GO, when exposed to aqueous environments, breaks down into a substance that is essentially dirt. This discovery positioned GO as a highly promising biomaterial, as it suggested that similar degradation could occur in vivo, given the aqueous nature of biological systems. However, it also raised concerns about the toxicity of the degradation products and the as-synthesized materials.

### 2.1 Early In Vitro and In Vivo Studies

**Figure 2:**
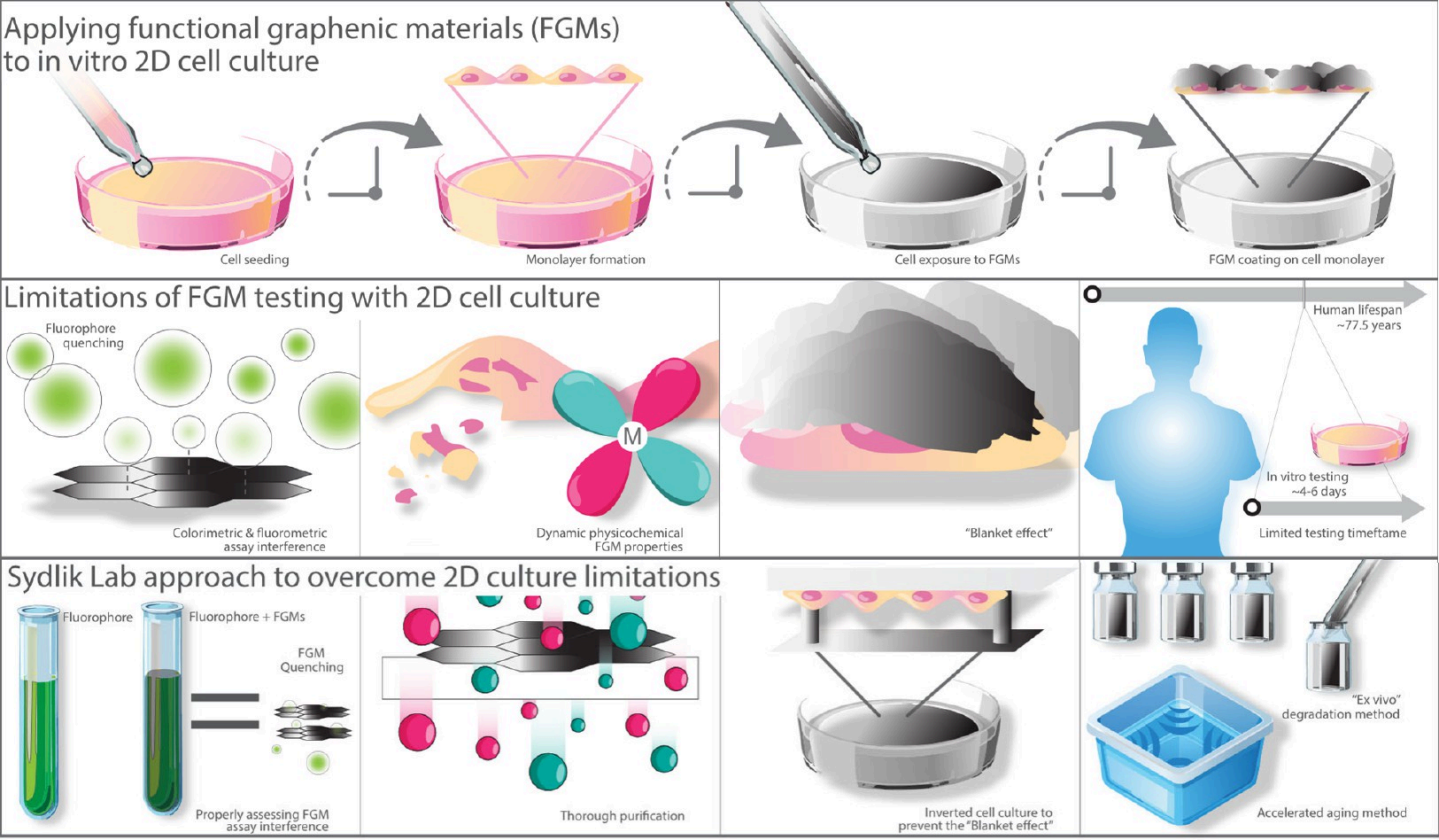
Overview of functional graphenic material (FGM) testing in 2D cell culture and the Sydlik lab’s strategies to address common limitations. The first row depicts the application of FGMs in a traditional 2D cell culture setup with adherent cells. The second row highlights limitations of this approach, such as interference with fluoro- and colorimetric assays due to adsorption to FGMs, resulting in quenching; dynamic physicochemical properties, such as impurities that affect compatibility; the “blanket effect” where FGMs cover and smother cells; and limited testing timeframes that fall short of mimicking human lifespans. The final row illustrates solutions from the Sydlik lab, including running proper assay controls to assess FGM interference, purifying and characterizing FGMs to address property variability, employing inverted cell culture to avoid the blanket effect, and using ex vivo degradation along with accelerated aging techniques like sonication to simulate long-term compatibility.

The early testing of GO compatibility centered on evaluating its impact on biological systems, focusing primarily on dose- and time-dependent exposure in both in vitro and in vivo models (Figure 2).^34,35^ In vitro studies explored GO’s effect on a variety of cell lines, including human fibroblasts,^36^ erythrocytes,^37^ and neuronal cells,^38^ assessing cytotoxicity, oxidative stress, and membrane disruption.^36−38^ These studies, among others, revealed that GO’s compatibility varied significantly depending on concentration, exposure duration, and cell type.^34,35^ Concurrently, in vivo testing predominantly involved mouse model studies,^34^ the gold standard for translating toxicity findings into human applications.^39^ Initial research focused on GO injections and their exposure to the bloodstream, aiming to understand how it interacted with tissues and organs.^34^ However, as the field progressed, several challenges emerged in both in vitro and in vivo models, highlighting the complexities of assessing graphene oxide’s biocompatibility and toxicity.

#### 2.1.1 In Vitro Compatibility of GO

Early cytocompatibility studies on graphene oxide focused on assessing its potential to induce cell death or apoptosis, often through mechanisms such as oxidative stress, membrane disruption, or other toxicity pathways. However, the findings on GO’s cytocompatibility were inconsistent. Some studies found that GO at doses above 20 μg/mL led to apoptosis and loss of adhesion in human lung fibroblasts within 24 hours, while lower doses had minimal effects.^40^ Other research showed that even at concentrations as low as 10 μg/mL, GO could cause mitochondrial damage and apoptosis in neuronal cells, with more pronounced effects at higher doses.^41^ In contrast, studies with human lung epithelial cells found no significant cytotoxicity, indicating that GO’s toxicity may depend on factors like cell type, or the specific form of graphene used.^42^ Furthermore, research on GO’s interaction with human red blood cells revealed that its aggregation state played a key role, with larger, aggregated sheets displaying lower hemolytic activity compared to more dispersed forms of GO.^37^ As the body of literature on GO’s cytocompatibility expanded, disagreements over its safety continued to grow, with findings varying widely depending on experimental conditions and methodologies.

#### 2.1.2 In Vivo Compatibility of GO

While in vitro studies provide foundational insights into GO’s compatibility, in vivo studies offer a more comprehensive view of how these materials interact with biological systems over time. Importantly, the intended application plays a crucial role in determining whether FGMs can be safely used. For instance, intravenous injection or inhalation of FGMs has been shown to cause severe toxicity or death in animal models due to aggregation in lung tissue.^43−45^ However, this aggregation-induced toxicity does not necessarily imply that the materials are inherently toxic. Many biomedical materials that are safe in one application can be harmful in others. For example, materials used in medical implants may be nontoxic when integrated into the body but could cause harm if injected or inhaled.

**Figure 3:**
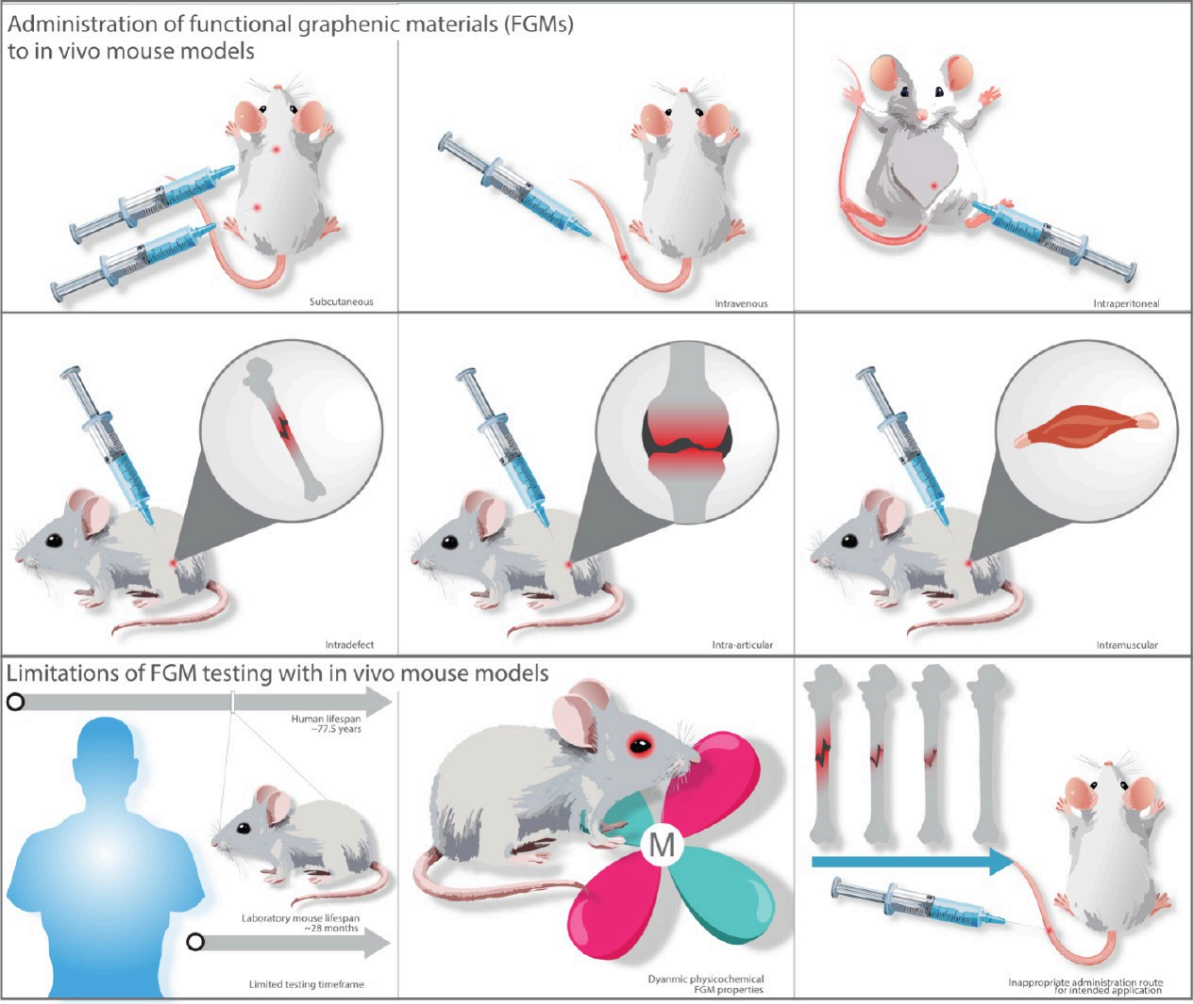
Overview of functional graphenic material (FGM) administration routes in in vivo mouse models and associated limitations. The first section illustrates common routes for FGM delivery, including subcutaneous, intravenous, intraperitoneal, intradefect, intra-articular, and intramuscular administration. The second section addresses the limitations of using in vivo mouse models for FGM testing, such as the discrepancy between mouse and human lifespans, dynamic physicochemical properties of FGMs, such as impurities that lead to artificial toxicity, and the potential for inappropriate administration methods that may not align with the intended therapeutic application.

Our studies on the in vivo compatibility of GO have shown promising results. In a mouse model, we evaluated GO with different oxidation levels in subcutaneous and intraperitoneal tissue sites—both of which are relevant for medical device applications (Figure 3).^46^ Our findings indicate that GO is moderately compatible in these environments, eliciting a typical foreign body reaction. A lower degree of GO oxidation was found to accelerate immune cell infiltration, uptake, and clearance, particularly in the peritoneal and subcutaneous sites. This suggests that surface modification strategies could be employed to further reduce the inflammatory response and improve the overall compatibility of GO for long-term medical device use.

Other studies support the role of surface modification in improving GO’s biocompatibility. Radiotracer techniques have shown that GO predominantly accumulates in the lungs at high doses, causing inflammation and granuloma formation, while lower doses exhibited no significant pathological effects.^47^ Functionalization of GO, such as with DOTA for radiometal chelation, altered its biodistribution, leading to significant urinary excretion and spleen accumulation without lung retention.^48^ These findings demonstrate how surface chemistry can influence pharmacokinetics and reduce toxicity.

Further evidence highlights the ability of thin, individualized GO sheets to undergo extensive urinary excretion without causing nephrotoxicity. Even at high doses, no damage to kidney structures or impairment of function was observed. In vitro studies revealed that kidney cells exposed to GO recovered their barrier function within 48 hours.^49^ These results emphasize the importance of dose, thickness, and functionalization in tailoring GO for biomedical applications.

Collectively, these findings underscore that while GO shows promise for applications like drug delivery, medical devices, or tissue engineering, its safe use depends on optimizing its properties and application-specific conditions to balance therapeutic efficacy and biocompatibility.

### 2.2 Challenges, Innovations, and Considerations in Toxicity

Assessing the cytotoxicity of graphene oxide presents a range of challenges that complicate the interpretation of compatibility results (Figure 2). Initial assay-based evaluations have revealed significant limitations, particularly in the use of colorimetric and fluorometric methods, which can be influenced by GO’s unique properties. Furthermore, the dynamic physicochemical characteristics of GO—such as flake size,^50,51^ oxidation state, chemical functionalization,^51^ and material purity^51−53^—add layers of complexity that often go unreported in compatibility studies. The “blanket effect”, a phenomenon identified in our research, further complicates traditional 2D cell culture assessments by artificially reducing cell viability at higher GO concentrations. Additionally, the limited timeframes of in vitro studies restrict the ability to fully understand the long-term effects of GO exposure. Together, these factors highlight the need for more comprehensive methodologies in cytotoxicity testing, which will be explored in the following sections.

#### 2.2.1 Assay Interference: A Key Challenge in Evaluating GO Cytocompatibility

One of the first major challenges in evaluating graphene oxide cytocompatibility arose from the interference of GO with standard viability assays used in in vitro testing. Early studies, which often relied on colorimetric and fluorometric methods like MTT and CCK-8, produced conflicting results regarding GO’s toxicity.^54^ The unique physicochemical properties of GO, including its large surface area, redox activity, and low opacity, were found to interfere with these assays by creating artifacts. For example, GO could reduce the assay signal, giving the false impression of decreased cell viability or heightened cytotoxicity. This issue highlighted a critical flaw in relying solely on conventional assays for assessing GO’s biocompatibility, as the material itself could distort the data, leading to inaccurate toxicity evaluations. As our research on functional graphenic materials advanced, we found that control assays were particularly valuable in identifying compatibility tests that were not artificially influenced by the FGMs themselves.

#### 2.2.2 Physicochemical Properties: An Underlying Source of Conflicting Cytotoxicity Reports

As the body of literature on graphene oxide (GO) cytocompatibility grew, it became evident that assay interference was not the only issue contributing to the conflicting reports of GO toxicity. The dynamic physicochemical properties of GO itself, such as lateral flake size, number of layers, oxidation state, chemical functionalization, presence of impurities, and overall acidic nature, all significantly affect cytocompatibility. Many of these variables are often overlooked or inadequately reported in studies evaluating GO. For example, the acidic nature of GO can create a cytotoxic environment if media changes are not performed frequently enough, leading to artifacts in the data. This toxicity is not a direct result of GO but stems from improper experimental design. Similarly, variations in flake size or oxidation state can dramatically influence how GO interacts with cells, leading to inconsistencies in results across different studies. This complexity makes it difficult to establish a clear consensus on GO’s biocompatibility, as many critical factors are not consistently controlled or documented.

In addition to complexities arising from physical characteristics of GO, chemical functionalization also plays a critical role in determining its biocompatibility. We have demonstrated that the functionalization of functional graphenic materials through the grafting of polypeptides onto the graphenic backbone not only enhances cytocompatibility, but also promotes cellular adhesion on the material’s surface. This polypeptide-grafted FGM shows a promising ability to interface with cells, suggesting its potential in applications requiring enhanced cell-material interactions.

Moreover, our work with phosphate graphenes (PGs)—a family of graphene-based materials functionalized with covalently attached polyphosphates—provides further insight into how chemical functionality can direct cellular behavior. The ionically balanced cations, such as calcium, magnesium, lithium, sodium, or potassium, in the PGs contribute to the material’s cytocompatibility in vitro. More importantly, these PGs are cell-instructive, releasing signaling ions that promote stem cell differentiation into bone cells, both in vitro and in vivo. This dual functionality of cytocompatibility and cell-instructive behavior highlights the potential of chemically modified graphenic materials for tissue engineering and regenerative medicine applications.

#### 2.2.3 The “Blanket Effect” and Limitations of Traditional 2D Cell Culture

Another critical factor complicating the evaluation of graphene oxide cytocompatibility is the use of traditional 2D cell culture setups, which can introduce an artificial decrease in compatibility at higher GO concentrations due to a phenomenon we identified as the “blanket effect.”^55^ This occurs when GO flakes, driven by gravity, settle onto the cells adhered to the bottom of culture dishes, effectively blanketing them over time. The rate at which these flakes settle depends on their size, with larger flakes (tens of microns) settling within the first 2 hours and smaller flakes (a few microns) continuing to cover cells significantly by 16 hours. Given that most cell culture experiments span several days to weeks, this accumulation can lead to an artificial reduction in cell viability. This decrease is not due to the inherent toxicity of GO but arises from the limitations of the experimental design.

**Figure 4:**
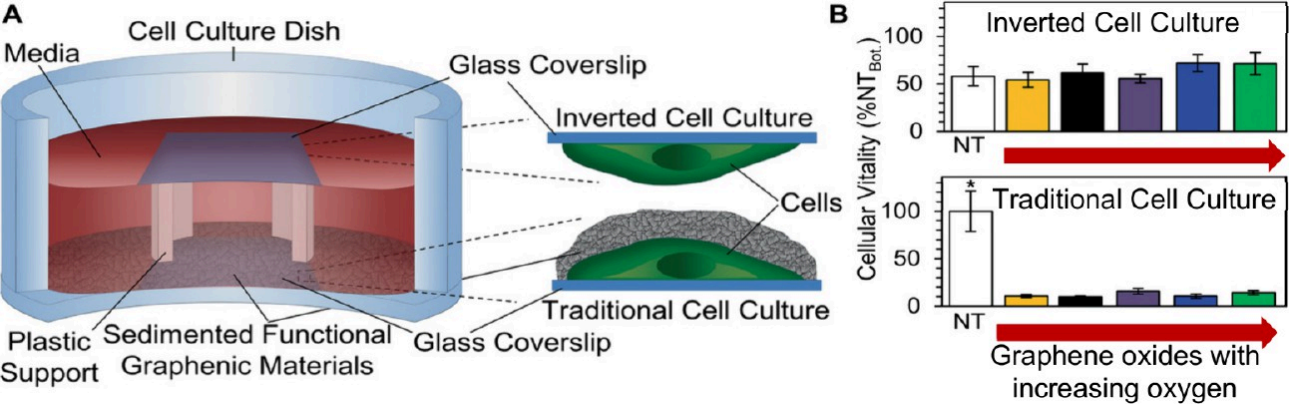
The “blanket effect.” (A) Graphical representation of our unique experimental cell culture setup and illustration of settling of FGMs. (B) Cellular vitality of cells cultured in inverted (top) and traditional (bottom) cell culture orientations. FGMs settle and artificially skew cytocompatibility results from traditional 2D cell culture. Data are mean ± standard error of *n* = 3 samples. ANOVA posthoc Tukey two-tailed *p* values <0.05 are indicated with # for Super GO compared to NT, GO3.4, GO1.8, and GO1.4 (top) and * for NT compared to all other conditions (bottom). Note that “NT” stands for no treatment. Reproduced with permission from ref (55). Copyright 2021 Wiley-VCH.

Our studies using an inverted cell culture technique, which mitigates the blanket effect, have demonstrated that GO is well-tolerated by fibroblast and macrophage cell lines (Figure 4). This finding highlights the importance of carefully considering the experimental setup when assessing GO toxicity. The blanket effect illustrates how the physicochemical properties of GO, combined with the limitations of typical 2D cell culture designs, can distort results and may not accurately reflect GO’s true biocompatibility in vivo.

#### 2.2.4 Work in the Sydlik Group to Overcome the Limitations of In Vitro Cytocompatibility Testing

While well-designed, traditional in vitro testing methods provide valuable insights into the cytocompatibility of graphene oxide, they are often limited by short timeframes, typically lasting only days to weeks. This restricted duration fails to capture the long-term effects of GO exposure, particularly as the material degrades or undergoes compositional changes. To address this limitation, we developed an ex vivo method that allowed us to study the cytocompatibility of GO and its degradation products over extended periods—up to 8 months—with human fibroblasts and macrophages (Figure 2).^56^ This approach is critical because it bypasses the temporal constraints of traditional in vitro cell culture testing. Our findings revealed that while GO does change compositionally as it ages in an aqueous environment, these changes do not negatively impact cellular vitality. Even as GO degrades and its structure evolves, it remains cytocompatible, causing no significant alterations to subcellular structures or DNA integrity.^56^

GO degradation not only involves changes in its structural and chemical composition but also leads to the evolution of specific degradation products, including volatile organic compounds and CO_2_, which typically emerge as gases.^57^ These degradation pathways are important to consider, as the release of volatile organics and CO_2_ could influence the local cellular microenvironment, particularly in confined or encapsulated biomedical applications. Future work will need to evaluate the effects of these gaseous byproducts to ensure their compatibility and safety for long-term use.

Despite the efficacy of ex vivo aging, the extended timeframes required can hinder rapid assessment of GO’s long-term stability and cytocompatibility, slowing down the research process. As in other areas of material science, accelerated aging techniques are particularly useful for overcoming this limitation, allowing for faster evaluations that can predict long-term outcomes.^58,59^ To this end, we developed an accelerated aging technique, inducing rapid degradation of GO through sonication in water. This method significantly reduces the time scale needed to evaluate GO’s stability and cytocompatibility, providing a more efficient approach for predicting long-term outcomes.^56^ We have since applied this accelerated aging technique to study other functional graphenic materials, broadening our understanding of their potential for safe, long-term use in biomedical applications, such as implanted tissue-engineered scaffolds.^60,61^

#### 2.2.5 Effect of GO on Immune Cells and the Immune System

Beyond simple compatibility, scientists must also be aware of the effects that GO, FGMs, and their degradation products have on the immune system.^62^ Our early work in 2015 evaluated the effect of GO oxidation state on cytocompatibility and found that more oxidized GO elicited a more inflammatory immune response, while reduced GO resulted in faster immune cell infiltration, uptake, and clearance.^63^ This is a similar result to work from another group, which found that PEGylation and other hydrophobic surface groups (carboxylic acids) elicit an elevated immune response.^64^ Newer FGMs should be designed with these findings in mind. Additionally, the interactions between FGMs and the immune system are two-sided, meaning that while GO elicits an immune response, cellular uptake and enzymatic activity must also be considered.^65^ Specifically, GO can be enzymatically degraded,^66^ and as more nanocarbon materials encounter the environment, adaptation to allow for more specific enzymes could be expected. Thus, it is prudent to consider enzymatic and biodegradation products of GO and FGMs, as well as any harmful, lasting effects on the immune system.

## 3 GO as a Biomaterial

### 3.1 GO as a Tissue Regeneration Scaffold

Researchers quickly recognized the versatility and utility of graphene oxide in tissue regeneration, thanks to its unique physicochemical properties. GO’s large surface area, high mechanical strength, and ability to promote cell adhesion make it an ideal scaffold for tissue engineering. Additionally, its tunable chemical functionalization, porosity, and gradual degradation in aqueous environments^33^ further enhance its potential across diverse applications. GO has been explored for various tissue regeneration purposes, including bone and cartilage regeneration, where its mechanical properties impart structural integrity and support osteogenesis.^67,68^ In nerve and cardiac tissue engineering, GO’s conductivity helps support nerve signal transmission and heart muscle function.^67^ Its hydrophilicity and bioactivity also make it suitable for skin and wound healing,^67,69,70^ while its biocompatibility aids in liver tissue regeneration by supporting hepatocyte growth.^71^

The versatility of chemical modifications of GO has extended its use to vascular tissue engineering, promoting endothelial cell adhesion and growth to restore blood vessels.^72^ Additionally, it has been applied in dental tissue regeneration, offering mechanical strength and antibacterial properties to repair dental structures and prevent infections.^73,74^ GO has shown potential in corneal tissue engineering, promoting epithelial cell growth and maintaining transparency, which is essential for restoring vision.^75,76^ With its broad range of applications across multiple tissue types, GO continues to be a promising material in the field of regenerative medicine.

#### 3.1.1 Bone

The inherent strength, versatility, and tunable surface chemistry of GO made it a natural candidate for designing scaffolds to support bone repair. Recognizing its potential, we developed a new class of FGMs specifically tailored for bone regeneration. Our primary goal was to create phosphate graphene (PG) scaffolds that not only provide structural support at the site of injury but also gradually break down in a controlled manner as new bone tissue forms, aligning with the natural timeline of bone healing.

##### 3.1.1.1 Phosphate Graphene Using the Arbuzov Functionalization

**Figure 5:**
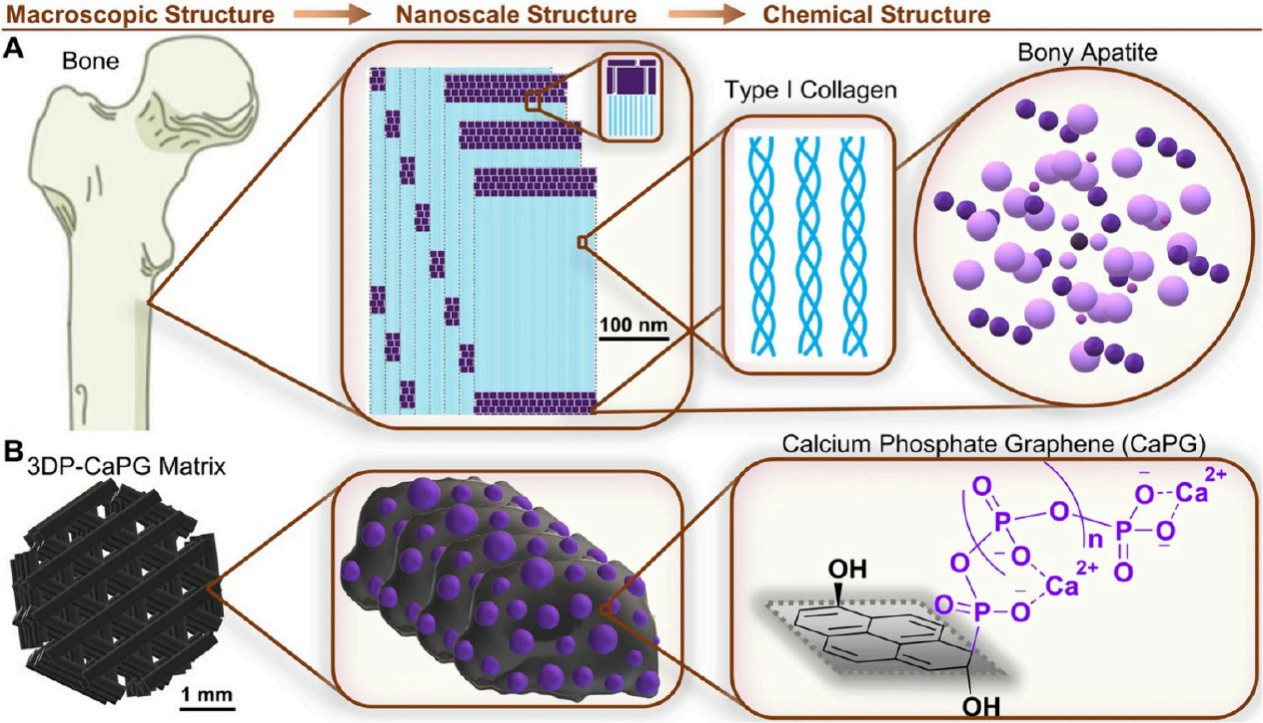
Biomimetic 3D printed CaPG matrix design. (A) Illustrative example of the structure of bone at the indicated length scales. (B) Illustration of the corresponding 3D printed CaPG matrix structure. Note that the graphenic sheet of CaPG is shown as a pyrene structure for simplicity. Reproduced from ref (80). CC BY 4.0.

The Arbuzov reaction can be used to install polyphosphate groups on the basal plane of graphene (Figure 5).^77^ This material was interesting when it was first synthesized because both the chemical composition and mechanical properties mimicked those of bone in our initial studies. However, this was before we began studies of compatibility, so we did not pursue the application at the time. However, after my postdoctoral work concerning compatibility and others’ work on the degradability of GO, the most promising early work in using FGMs for tissue regeneration in my group was for bone regeneration.

In my lab, we found that using a Lewis acid catalyst improved the reaction efficiency, allowing us to incorporate less reactive counterions into the phosphate structure. The original work used a lithium counterion, but with catalytic control, we were able to easily generate phosphate graphene with Li^+^, Na^+^, Mg^2+^, Ca^2+^, or K^+^ counterions.^17^ Initial in vitro experiments showed that calcium phosphate graphene (CaPG) was our star—CaPG was able to induce osteogenesis to the same extent as osteogenic media in vivo, without the use of any growth factors. Our studies demonstrated that this was because the material provided a sustained release of Ca^2+^ and PO_4_^3–^ ions. These ions are reported to be “inducerons,” or ions that are able to induce stem cell differentiation.^78^ CaPG was then implanted in vivo in mice and showed good results, using an ectopic model for bone growth. This work demonstrated CaPG is intrinsically osteoinductive in vivo, retaining and recruiting stem cells while directing osteogenic differentiation.^79^

##### 3.1.1.2 GO as a Material for Bone Regeneration

Many other researchers have recognized the potential of GO in bone regeneration. Even without the Arbuzov modification, GO has demonstrated an ability to significantly enhance and accelerate the osteogenic differentiation of mesenchymal stem cells.^81,82^ GO’s potential to induce bone formation around modified materials further solidifies its role as a versatile scaffold material in implant applications.^34,83^ Several studies have explored GO’s osteoinductive properties, providing a promising outlook for its use in bone tissue engineering and implant applications.

In one study, a rabbit tibia model was used to explore the effects of GO-coated titanium surfaces on osseointegration. GO demonstrated a notable improvement in the viability, proliferation, and osteogenic differentiation of bone marrow stromal cells (BMSCs). Additionally, GO-coated surfaces enhanced the attachment of human gingival fibroblasts and significantly improved osseointegration in vivo. This work suggests that modifying the physicochemical properties of implant surfaces with GO can stimulate bone healing at the bone–implant interface, providing a promising approach for orthopedic applications.^84^

Further comparative studies investigated polycaprolactone (PCL) scaffolds coated with graphene and graphene oxide, revealing GO’s ability to promote osteogenic differentiation in mesenchymal stem cells even without differentiation media. While graphene-modified scaffolds exhibited higher surface roughness, GO-coated scaffolds showed superior mineralization, suggesting a higher osteoinductive potential. This indicates that GO’s surface properties play a crucial role in bone regeneration and scaffold design.^85^

Free-standing GO foils have also been explored as substrates for stem cell differentiation. Dental pulp stem cells (DPSCs) grown on these foils exhibited increased expression of key osteogenic markers, including RUNX2 and SP7, and enhanced extracellular matrix production. This study further underscored GO’s ability to accelerate osteogenic differentiation and matrix synthesis, highlighting its potential to drive bone formation effectively.^86^

In addition to its osteoinductive capabilities, GO has been found to exert immunomodulatory effects, particularly when used on titanium-coated surfaces. By influencing macrophage polarization, GO was able to create a pro-osteogenic environment while reducing inflammation under acute conditions. This dual role of enhancing both osteogenesis and immune modulation makes GO an ideal material for modifying bone scaffolds and implants to promote bone healing.^87^

These findings, while only a snapshot of the extensive literature on GO and osteogenesis, underscore GO’s remarkable osteoinductive properties, positioning it as a leading material for bone tissue engineering and implant surfaces. Its dual ability to promote osteogenesis and modulate immune responses offers substantial advantages for applications focused on enhancing bone regeneration and improving implant integration.

### 3.2 GO and FGMs as Additives and Polymer Composites for Tissue Regeneration

Beyond bone regeneration, the versatility of GO has been harnessed in a variety of other biomedical contexts, including cardiac,^88−90^ nerve,^91,92^ cartilage,^93,94^ and skin regeneration,^95^ as well as wound healing. Its ability to be integrated with other bioactive materials through methods like coating, hydrogel blending, and 3D printing, allows for the engineering of complex, multifunctional constructs. These constructs can mimic the mechanical and electrical properties of native tissues while also providing a conducive environment for cellular growth and proliferation.

The hydrophilic and biodegradable nature of GO, combined with its unique mechanical, electronic, and chemical properties, makes GO an exceptional candidate for developing advanced biomaterials.^96^ Unlike graphene, GO’s hydrophilic oxygen-containing groups facilitate the formation of stable aqueous suspensions, reducing the risk of irreversible agglomeration and making it more suitable for biological applications.^97−99^ These oxygen-containing functional groups^15^ also enable GO to form covalent bonds, as well as hydrophobic, electrostatic, and hydrogen-bond interactions with various natural and synthetic polymers, a property that has been harnessed for designing composites.

#### 3.2.1 Peptide–GO Composites and FGMs

**Figure 6:**
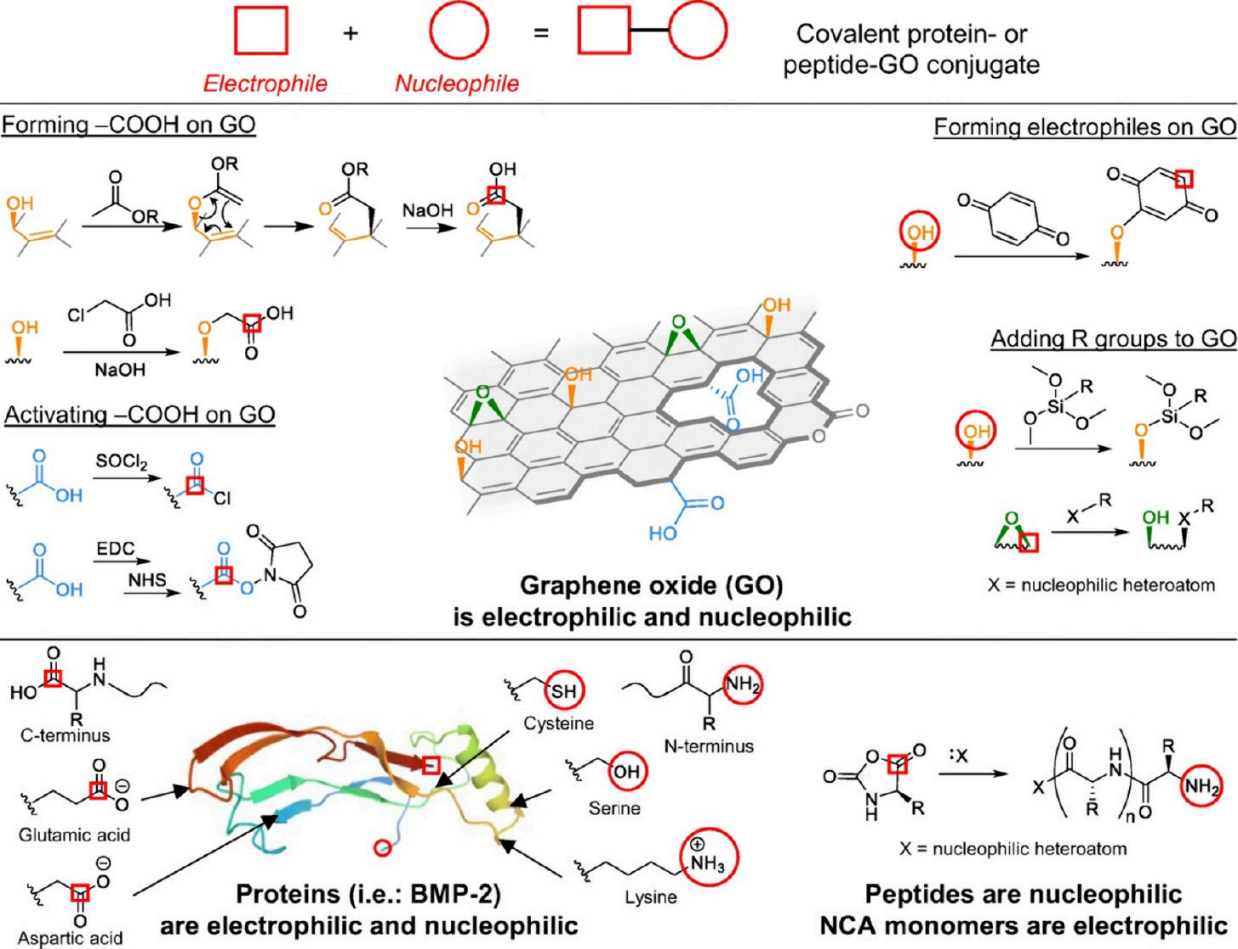
Peptides and proteins can be covalently conjugated to GO through a variety of synthetic routes. Note that the protein image was created using Protein Data Bank (PDB)^100^ entry 3BMP of bone morphogenic protein-2.^101^ Reproduced with permission from ref (102). Copyright 2020 The Regenerative Engineering Society.

Our group has used the Claisen modification of graphene^20^ to produce compatible FGMs for biomedical applications. Claisen graphene (CG) is a reduced form of GO and provides improved conductivity and cytocompatibility when compared to GO. To develop CG as a biomaterial, we developed methods for covalently grafting peptides, resulting in biocompatible and mechanically robust peptide-functionalized FMGs (Pep-G) (Figure 6).

**Figure 7:**
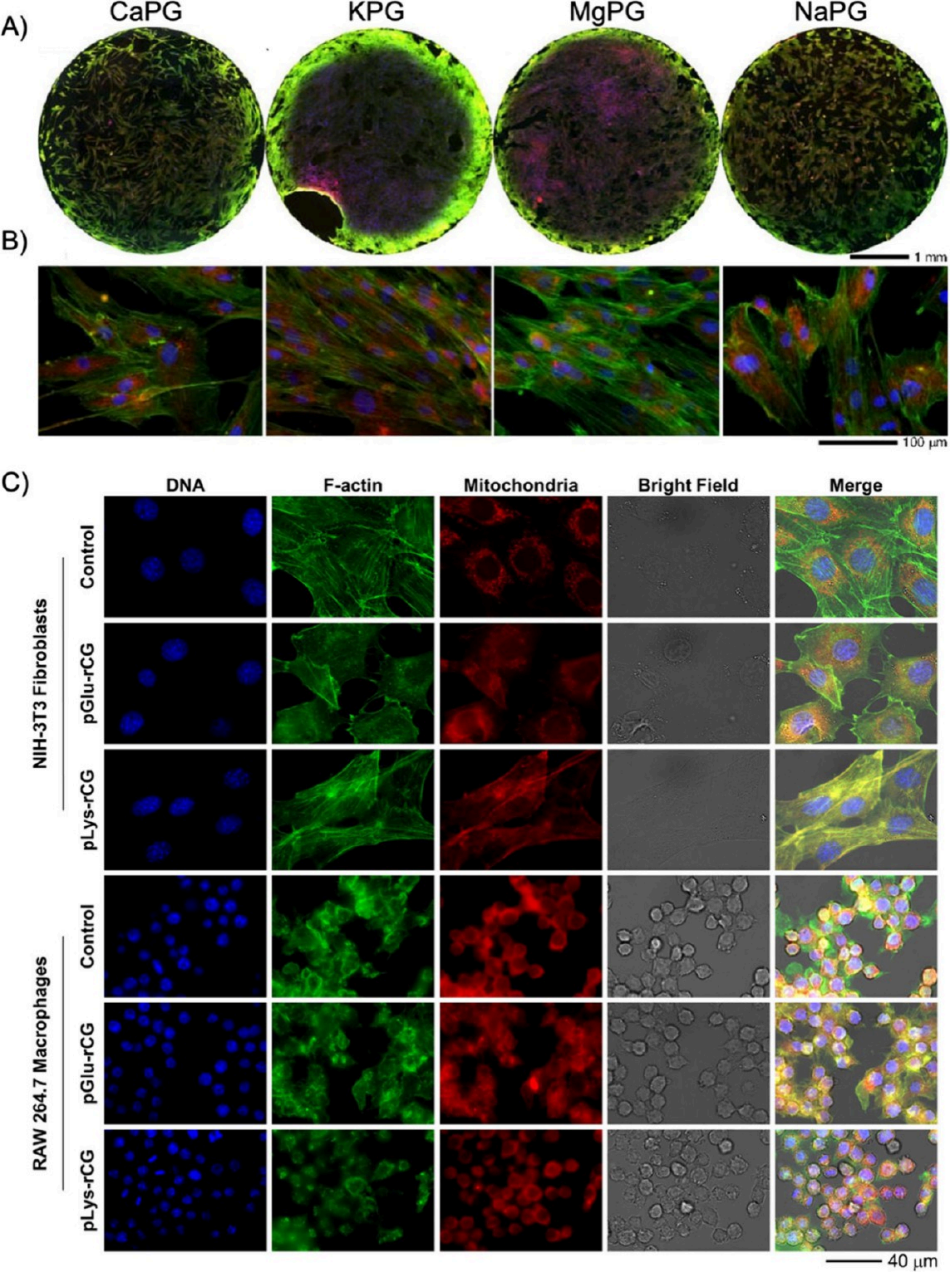
hMSCs adhere to and grow on 3D scaffolds of peptide Claisen graphene (Pep-G) materials. (A) Whole-pellet images (top view) and (B) higher-magnification images of hMSCs cultured on PG pellets for 7 days and then labeled for nuclei (blue), F-actin (green), and mitochondria (red).^79^ (C) rCG functionalized with poly(lysine) (pLys-rCG) and poly(glutamate) (pGlu-rCG) were found to be cytocompatible. Subcellular imaging of nuclei (DNA), cytoskeleton (F-actin), and mitochondria of NIH-3T3 fibroblasts and RAW 264.7 macrophages exposed to 50 μg mL^–1^ peptide-functionalized rCG for 1 day. There were no observed deleterious effects on these important subcellular compartments compared to untreated control cells.^103^ Reproduced with permission from ref (103). Copyright 2017 Society of Chemical Industry.

Initially, a grafting-from approach was used to create Pep-Gs. CG was further reduced, to transform carboxylic acids into primary alcohols, suitable for initiating the ring-opening polymerization of *N*-carboxyanhydride monomers from nucleophilic sites on CG.^103^ Although this approach produced promising cell scaffold materials (Figure 7A), we encountered difficulties in controlling peptide length and molecular weight dispersity due to the other nucleophilic groups on CG. In addition, while CG is somewhat reduced, some adsorbed water remains associated, which limits molecular weight through premature termination.

**Figure 8:**
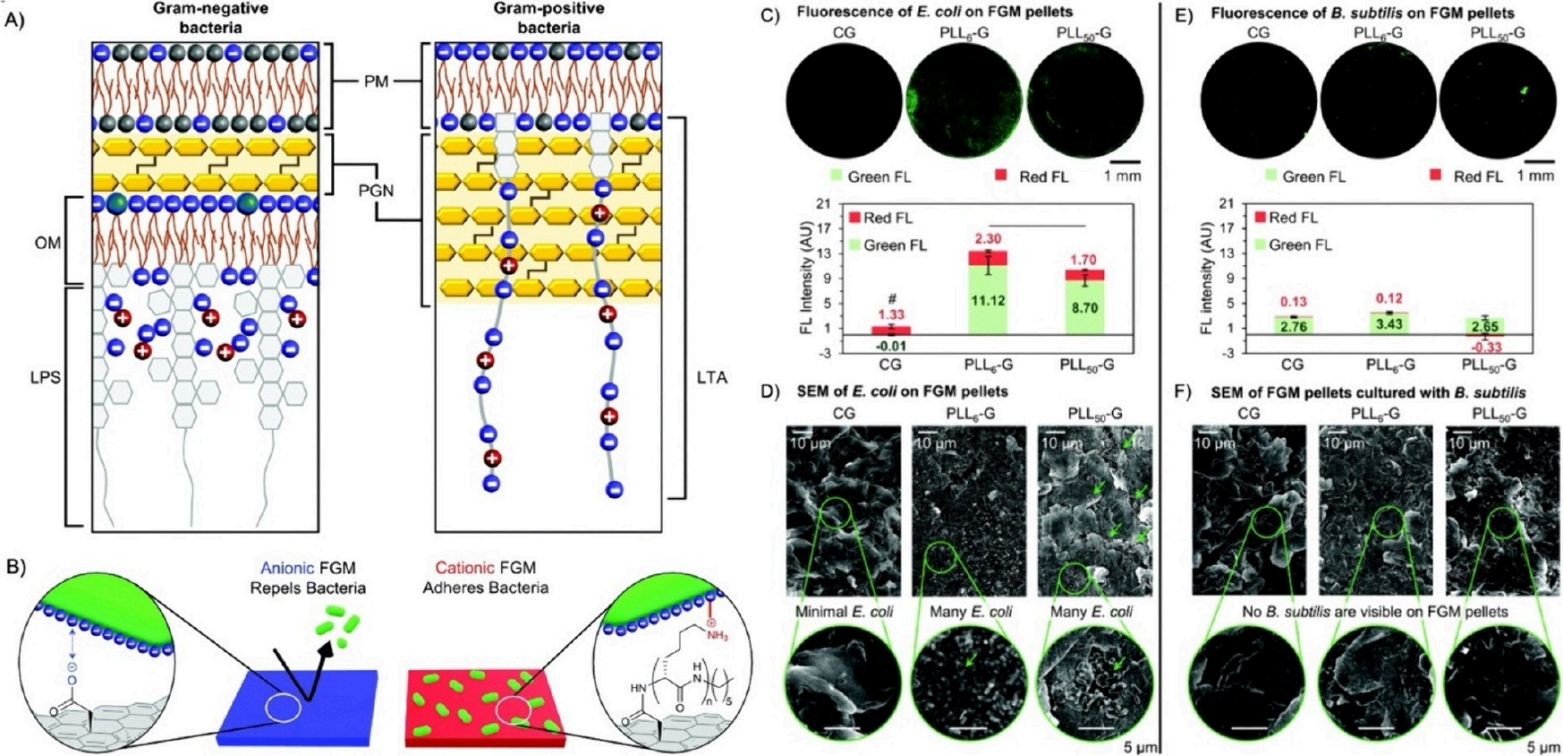
(A) Gram-negative and Gram-positive bacteria have different cell wall architectures (based on information from Malanovic and Lohner^1000^). PM = plasma membrane; PGN = peptidoglycan; OM = outer membrane; LPS = lipopolysaccharide; LTA = lipoteichoic acid. (B) The charge of a functional graphenic material (FGM) surface, which can be tailored in the synthesis of the material, can influence the interaction of the FGM with the net negative charge of the bacterial cell wall. (C) Fluorescence microscopy images of *Escherichia coli* adhered to the FGM pellets demonstrate that CG is bacterio-repellant, and the PLL_*n*_–G conjugates are bacterio-adhesive. Quantification of the green and red fluorescence reveals that CG has significantly fewer total cells (Green FL + Red FL) than either of the PLL_*n*_–G materials (*p* < 0.05, “#” symbol). Further, PLL_6_–G fosters more live cells (Green FL) than PLL_50_–G (*p* < 0.05, black bar). (D) SEM of FGM pellet surface following overnight culture with *E. coli*. Green arrows indicate regions on PLL_50_–G where bacteria are clustered. Select areas are enlarged to show bacteria morphology. (E) Fluorescence microscopy images of *Bacillus subtilis* adhered to the FGM pellets show few, localized bacteria on all FGMs. Quantification of fluorescence intensity reveals that all FGMs possess statistically equal amounts of adhered *B. subtilis* (*p* > 0.05 when comparing CG, PLL_6_–G, and PLL_50_–G pellet fluorescence intensities). (F) SEM of FGM pellet surface following overnight culture with *B. subtilis*.^104^ Reproduced with permission from ref (104). Copyright 2020 Royal Society of Chemistry.

To overcome these challenges, we developed an alternative end-capping synthetic strategy that allows for precise control over peptide identity and molecular weight. By first initiating the polymerization and allowing propagation to proceed in the absence of the FGM, we were able to control molecular weight and amino acid composition, terminating the polymerization with an electrophilic CG only when the desired molecular weight of the polymer was reached.^91^ Using this method, we conjugated homopeptides, including polylysine and polyglutamate (pLys-CG and pGlu-CG), as well as block copolypeptides. pLys-CG and pGlu-CG conjugates exhibit good cytocompatibility and are mechanically robust when processed into 3D constructs (Figure 7B). Notably, Pep-G constructs demonstrate enhanced conductivity compared to their parent graphenic materials (GO and CG), a feature critical for their intrinsic functionality in vivo. This characteristic was further validated in a preliminary nerve regeneration study, where electrically stimulated Pep-G pellets enhanced cell adhesion and neuronal-like differentiation in PC12 cells.^91^ This research introduces a promising class of Pep-G materials for regenerative tissue scaffolds. Moreover, the CG functionalization strategy holds significant potential in cell instructive materials, such as bacterio-instructive materials (Figure 8), which capitalize on interactions with the bacterial cell wall.^104^ Further, FGMs can be tailored to covalently attach virtually any bioactive moiety to CG, enabling the creation of FGMs that target specific healing pathways.

**Figure 9:**
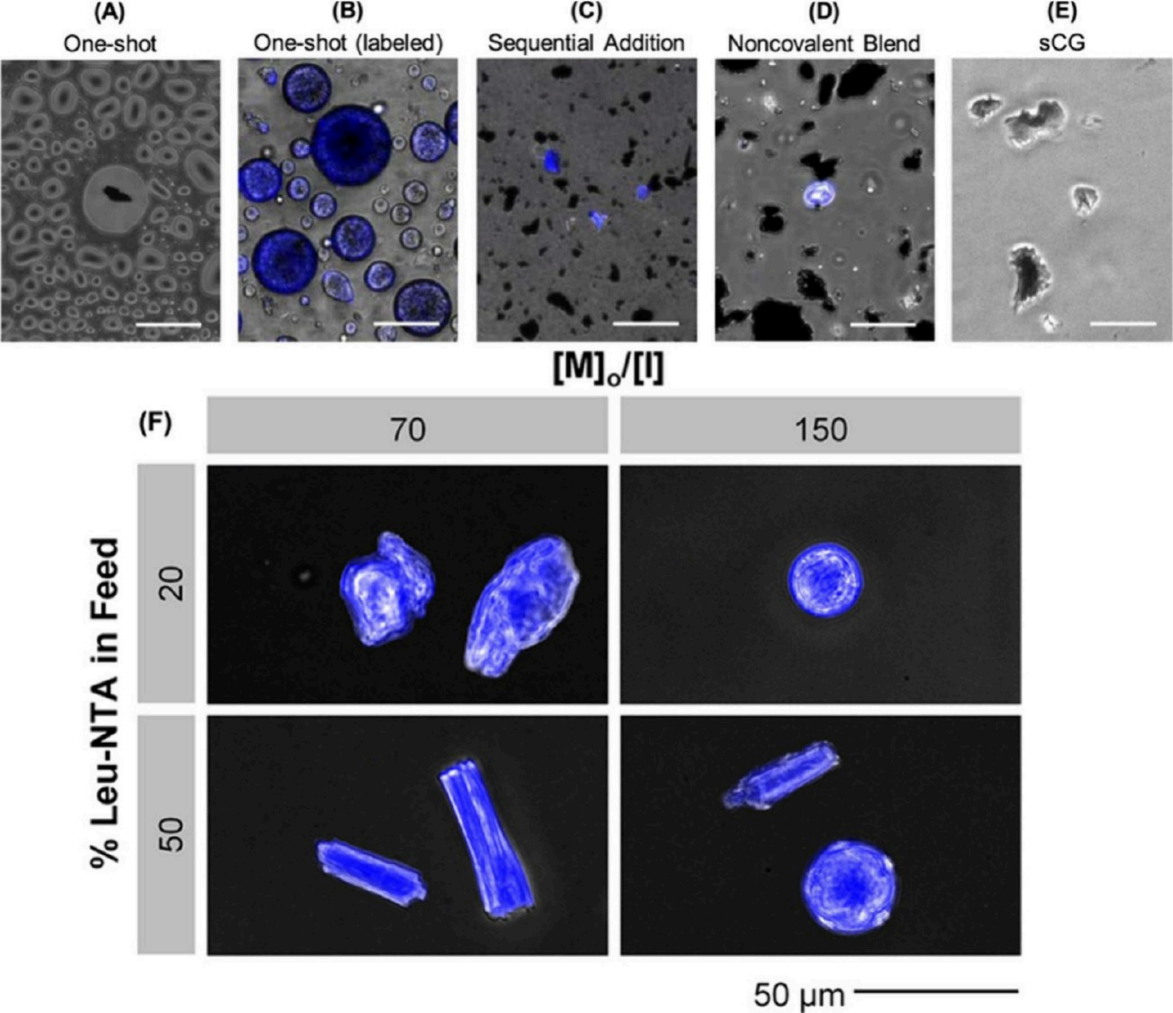
(A, B) One-shot peptide amphiphiles (PA) are end-capped with a graphenic material to give PA–G, where the PA templates the assembly of the graphenic material. (C) When PA is synthesized by sequential NCA monomer addition, end-capping with the graphenic material does not result in directed graphenic assembly. (D) Similarly, noncovalent blends of one-shot PA with the graphenic material result in no directed assembly. (E) Optical image of the pure graphenic material (sCG) prior to end-capping with PA is given for comparison of morphology. Scale bars represent 50 μm. Note that the graphenic material is black; in (B–D), the PA is labeled with FM-4-64 fluorescent dye (represented in blue). (F) One-shot peptide-amphiphiles (PAs) assemble at 1 wt % in a solution of 99% DI H2O and 1% TFA. PA assemblies, represented in blue, were visualized by staining with FM-4-64 fluorescent dye (excitation 558 nm, emission 734 nm). Reproduced from ref (105). Copyright 2020 American Chemical Society.

FGMs hold promise in biomedicine due to their biocompatibility, electrical conductivity, mechanical strength, and capacity for bioactive functionalization. However, these properties are best realized in ordered structures where graphenic sheets are aligned. Current bottom-up assembly methods for creating such ordered 3D structures are limited. To overcome this, we developed a strategy that templates the bottom-up assembly of FGMs by covalently binding a self-assembling polypeptide amphiphile (PA) to a graphenic surface, resulting in a PA–graphenic conjugate (PA–G). This was achieved by modifying our peptide end-capping procedure, which is more effective than traditional grafting methods due to reduced steric hindrance and interference from adventitious functional groups on the graphene. To accomplish this functionalization, PA was synthesized via a one-shot copolymerization strategy developed in-house,^105^ then end-capped with our electrophilic CG. Deprotection of the PA exposed positively charged amines, leading to the directed assembly of PA–G conjugate structures (Figure 9). Fluorescent dye assays confirmed that the graphenic material was encapsulated by a PA shell, demonstrating the successful directed assembly of the conjugates.^105^

##### 3.2.1.1 Other Works

Peptide-functionalized GO also holds promise in sensors.^106−110^ Attachment of the RGD peptide onto a graphene surface has been explored to develop a biomimetic film sensor with smart, responsive properties.^108^ The RGD sequence was chosen for its ability to mimic the cell-binding sequence of extracellular matrix proteins, promoting excellent cell adhesion. To prepare the sensor, graphene oxide was chemically reduced and simultaneously functionalized with pyrenebutyric acid, which adsorbs onto the graphene surface via π–π interactions. The pyrenebutyric acid-functionalized graphene was then assembled into a film using a direct filtration approach. The final biomimetic graphene-based film was constructed by covalently bonding the RGD peptide onto the surface of the pyrenebutyric acid-functionalized graphene film through EDC/NHS coupling chemistry. This sensor not only significantly enhanced cell adhesion and growth but also enabled real-time electrochemical detection of nitric oxide molecules released from human cells under drug stimulation.

A similar EDC/NHS coupling chemistry was employed to attach nisin, a 34-amino acid antimicrobial peptide, to the surface of 2D graphene oxide (GO).^111^ Amine-functionalized PEG was then used to create a 3D porous architecture by interconnecting GO with the antimicrobial peptide, resulting in pore sizes of 200–350 nm. This nisin-conjugated porous 3D GO membrane was reported to enable the separation, identification, and complete disinfection of multidrug-resistant pathogens, such as MRSA, from water. The membrane’s pore size (∼300 nm), which was significantly smaller than MRSA (∼1000 nm), allowed only water to pass through while capturing the pathogens. The membranes demonstrated 100% removal and killing of MRSA. The nisin–GO-conjugated membrane dramatically enhances MRSA destruction through a synergistic effect compared to nisin and GO alone. The 3D GO structure traps MRSA, inducing membrane stress that facilitates nisin binding to the bacterial lipid II and phospholipid membrane, resulting in the effective elimination of nearly all the bacteria.

Covalent conjugation of BMP-2-like polypeptide onto GO has also been successfully attempted using similar EDC/NHS coupling chemistry. Using EDC and NHS as coupling agents, the carboxyl groups on GO were covalently linked to the amino groups of the peptides, forming a stable GO–peptide conjugate.^112^ This functionalized GO was then integrated into chitosan-coated silk fibroin electrospun scaffolds through electrostatic interactions, which not only enhanced the biocompatibility of the scaffolds but also ensured the sustained release of the peptides. The GO–peptide conjugate effectively prolonged the half-life of BMP-2, preventing its rapid deactivation in vivo. In a critical cranial defect rat model, these GO-BMP-2 scaffolds demonstrated significant bone regeneration, highlighting the potential of GO-functionalized scaffolds in tissue engineering. By the 21st day, approximately 49.43% ± 2.93% of the BMP-2 peptide was released, indicating that GO functionalization supports continuous and steady peptide release, improving cellular interactions and reducing the risk of rapid diffusion.

#### 3.2.2 Functionalization with Proteins and Nucleic Acids

##### 3.2.2.1 Protein-Functionalized GO

Functionalizing GO with proteins, such as bone morphogenetic protein-2 (BMP-2), can greatly enhance the stability and biological effectiveness of these biomolecules. Indeed, our group loaded GO with BMP-2 noncovalently as a positive control in our 2019 PNAS paper.^79^ Electrostatic interactions have also been employed to load BMP-2 on negatively charged GO-carboxylate surface with an aim to deliver BMP2 protein from a GO-coated titanium substrate.^113,114^ This resulted in a significant increase in vitro in alkaline phosphatase expression, a key marker of bone formation. In vivo, this system also accelerated new bone formation compared to those without GO. Notably, using GO to deliver BMP-2 reduced the required dose by half compared to conventional delivery methods. GO not only ensures the sustained release of BMP-2 but also enhances the protein’s structural stability and bioactivity.

While adsorption is the common method for protein-GO conjugation, covalent attachment offers better control over release kinetics, preserves hydrophobic regions for other materials, and minimizes denaturing interactions by targeting hydrophilic regions.^102^ Covalent reactions must be mild to avoid protein denaturation and selective to prevent unwanted side reactions.^102^ In one example, alkaline protease was covalently immobilized on GO sheets using glutaraldehyde. GO was first modified by glutaraldehyde followed by addition of alkaline protease leading to covalent interactions.^115^ Another study described the covalent immobilization of BSA on GO sheets through adiimide-activated amidation reaction under ambient conditions in water.^116^

Another study demonstrated the unique potential of GO as a protein delivery carrier in promoting chondrogenic differentiation of stem cells in a 3D culture system.^93^ Unlike conventional approaches using 2D culture substrates, this study utilized GO as a protein-delivery carrier to address challenges such as low cell-ECM interaction and the diffusional limitations of protein transforming growth factor-β3 (TGF-β). The distinctive surface chemistry of GO, characterized by hydrophobic π-domains and hydrogen bonding carboxylic and hydroxyl groups, enabled robust adsorption of fibronectin and TGF-β3 through π–π and electrostatic interactions. This stable adsorption facilitated sustained delivery of TGF-β3 and enhanced cell-adhesion, leading to significantly improved chondrogenic differentiation of human adipose-derived stem cells (hASCs).^93^ The findings underscore GO’s potential as a powerful tool in tissue engineering, offering a novel approach to overcoming the limitations of conventional stem cell differentiation methods.

##### 3.2.2.2 Nucleic Acid GO

**Figure 10:**
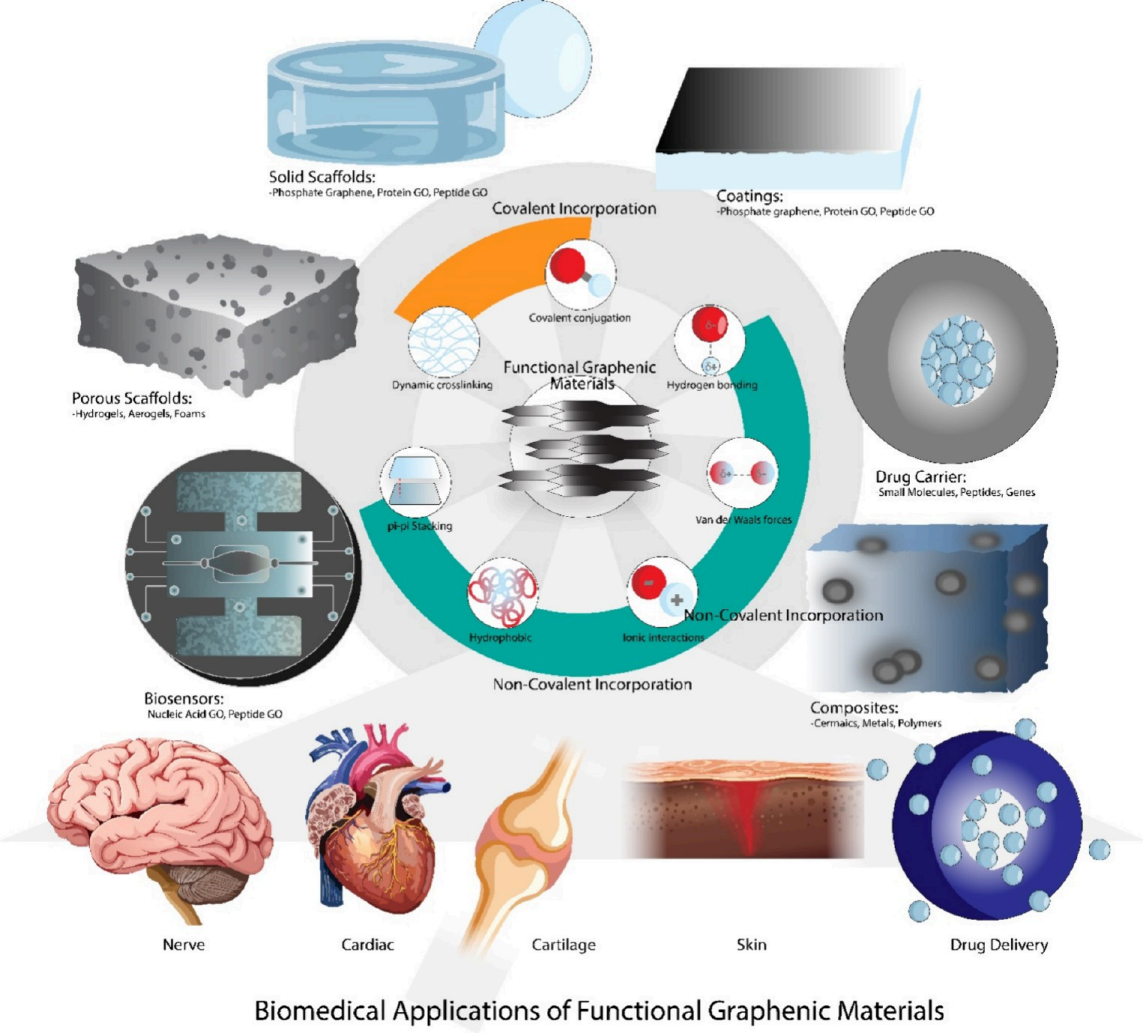
Overview of functional graphenic material (FGM) formulations, their major interaction types, and application areas.

Another important class of natural biomacromolecule is nucleic acids, and these have also created conjugates with GO. For example, a microRNA (miRNA) sensing platform was developed using dye-labeled peptide nucleic acid (PNA) and nanosized GO (NGO) for the real-time, sensitive, and quantitative monitoring of multiple miRNAs in living cells.^117^ The sensor lights up when quenched fluorescence of dye-labeled PNA is recovered upon the addition of target miRNA. PNA is a non-natural nucleic acid analog and has uncharged amide bonds in its backbone, in contrast to the negatively charged phosphodiester bonds in DNA. The use of PNA as a probe and NGO as a fluorescence quencher in this sensing strategy provides high selectivity and specificity for target miRNAs with minimal background signal and low cytotoxicity. PNA is an ideal candidate for conjugation with GO because PNA is inherently more stable than DNA or RNA, with high target specificity that is further stabilized by the GO substrate.^118^ Taken together, FGMs can be functionalized with a variety of biomolecules and incorporated for numerous applications (Figure 10).

#### 3.2.3 Challenges Limiting Clinical Application

Graphene oxide (GO) and functional graphenic materials (FGMs) hold significant promise as materials for regenerative applications, particularly in tissue regeneration. However, several major challenges have slowed the translation of these materials to the clinic.

##### 3.2.3.1 Characterization

Like polymers, characterizing functional graphenic materials (FGMs) presents inherent challenges due to their size and dynamic nature. This dynamic behavior arises from the diverse chemical environments present throughout the material. For example, the chemical environment of alcohol groups can differ depending on their location within the FGM structure, leading to peak broadening in analytical methods like spectroscopy, gravimetric, and thermal techniques. This makes the identification and quantification of functional groups complex.

To gain a more complete understanding of FGMs, they often need to be characterized by a suite of analytical techniques, with each offering only a small piece of the puzzle. Analyzing these pieces requires extensive experience in interpreting the data from individual methods. On top of this, assembling the various puzzle pieces to create a coherent picture of the material’s chemical properties also demands a high level of expertise. This combination of factors makes the learning curve for FGM characterization quite steep, requiring both technical proficiency and interdisciplinary knowledge.

In addition, FGMs are not compatible with most analytical instruments. Any instrument that requires FGMs to be injected poses a risk of damaging the equipment. For example, it is not advisible to use gel permeation chromatography (GPC) or any column-based instrument with FGMs, due to their inherent tendency to aggregate and adhere to surfaces, potentially clogging the instrument. Cleaning an instrument after exposure to FGMs can be extremely labor-intensive, and in some cases, complete replacement of the instrument may be necessary.

##### 3.2.3.2 Heterogeneity of Structure

Another layer of complexity in the characterization of FGMs arises from their inherent heterogeneity. The physicochemical properties of FGMs can vary significantly during both synthesis and purification, even within the same batch of materials, let alone across different batches. Variations in particle size, the number of graphene layers, oxidation degree, and functionalization density often occur, creating subpopulations of FGMs that may behave very differently from one another, particularly in in vivo environments. This heterogeneity poses challenges for assessing the safety and efficacy of FGMs, as materials that differ in structural attributes can exhibit widely varying biological responses. Furthermore, tracking carbon-based nanomaterials in in vivo systems is particularly difficult, as they tend to blend into the carbon-rich biological tissues, complicating efforts to monitor their biodistribution and clearance. These challenges in both heterogeneity and in vivo tracking have proven to be significant hurdles in accurately assessing the toxicity of FGMs, thereby impeding their approval for use by regulatory bodies such as the FDA and other global agencies.^119^

##### 3.2.3.3 Bulk Processing

A major obstacle to realizing their potential in biomedical contexts is the processing of these materials into suitable three-dimensional (3D) constructs because they are powders. For FGMs to be viable in tissue regeneration, they must be transformed from loose powders into customizable scaffolds that can be tailored to the specific anatomy of individual patients. As an example from my own research, PGs have presented challenges for. PGs have demonstrated osteoinductive and osteoconductive properties both in vitro and in vivo. Although our in vivo studies with PGs were promising, they resulted in poorly defined, amorphous bone formations rather than precise bone structure.

Initially, we used hot pressing to convert FGMs from powders into disc-shaped structures to be used in cell culture. While the mechanical properties of these structures were excellent, we found that it has several significant limitations: (1) Porosity: Hot pressed scaffolds do not offer necessary porosity for adequate cell infiltration. The ability to control the internal architecture—such as pore sizes and distribution—is limited, hampering the versatility needed for diverse applications. (2) Fixed structure: The hot-pressing method results in rigid structures with predetermined geometries, which do not allow for the customization required for specific biomedical implants. (3) Brittleness: After hot pressing, the bulk scaffold created is strong and holds robust mechaical properties. However, machining the construct to tailor the shape is not possible. Upon machining, FGMs constructs often crumble, which is unsuitable for further processing. These limitations in materials processing pose challenges in achieving the precise shapes and structural integrity necessary for functional implants. These limitations underscore the urgent need for more advanced processing techniques, such as 3D printing, to fully leverage the potential of GO and FGMs in creating tailored, functional biomedical implants.

### 3.3 FGM incorporation into bulk materials and scaffolds using 3D Printing

As powders with interesting mechanical and electronic properties, FGMs are a natural choice as a filler material for polymers. Thus, after incorporation into the composite, all processing methods used for polymer processing can be considered. Additive manufacturing, namely 3D printing, is an attractive processing method due to its customization, speed, waste reduction, and low cost,^120^ all attractive features for processing FGMs.

#### 3.3.1 Polymeric binders

Traditional methods, such as hot-pressing PGs into disc-shaped constructs, allowed us to assess important properties like cell adhesion and cytocompatibility. However, these methods were limited by their inability to control internal structures, such as porosity, which is essential for nutrient flow and tissue ingrowth. Additionally, the bulk materials produced were often too soft for further machining, complicating their adaptation into functional, patient-specific implants.

To overcome these limitations, we turned to 3D printing as a promising solution. Additive manufacturing enabled us to design patient-specific, three-dimensional constructs tailored to the site of injury, addressing the critical need for customizable geometry and internal structures. Advances in this technology have demonstrated the potential of incorporating graphenic materials into scaffolds for bone regeneration. However, previous studies primarily used unfunctionalized graphene, which lacks the osteoinductivity required for effective bone healing, and the amount of graphenic material included in these constructs was typically low.

In response to these challenges, we developed a new family of 3D printed phosphate graphene (3DP-CaPG) matrices, which incorporate a high weight fraction (90% w/w) of functional graphenic materials.^80^ This high concentration ensures that the bioactive properties of PGs, such as the controlled release of calcium and phosphate ions, dominate the biological response rather than being overshadowed by the bioinert binder used in most scaffolds. These 3DP-CaPG matrices are mechanically resilient and customizable while being inherently osteoinductive, promoting the differentiation of stem cells into bone cells both in vitro and in vivo.

**Figure 11:**
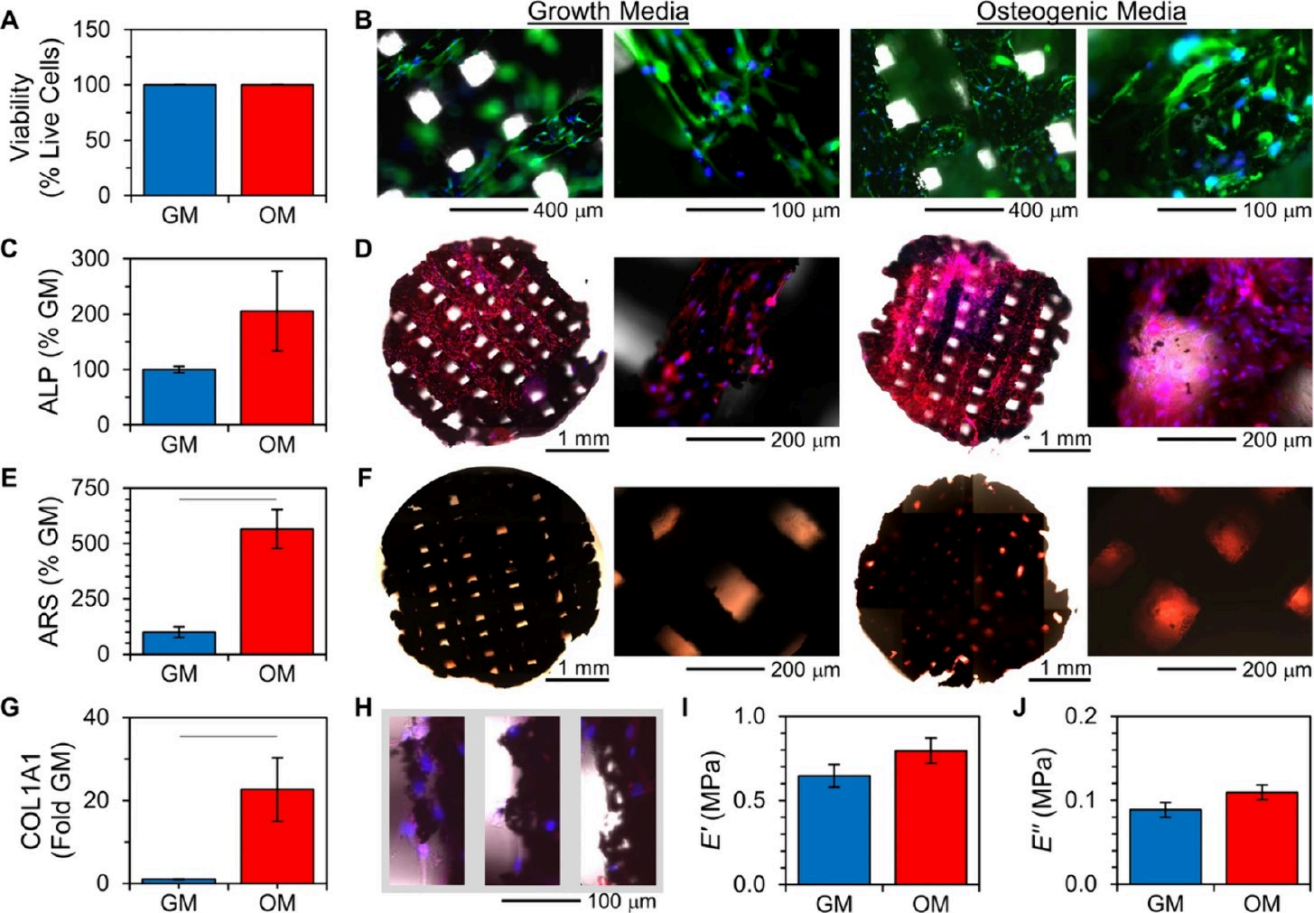
Compatibility and osteogenic differentiation of hMSCs on 3DP-CaPG matrices. (A) Percent cellular viability. *n* > 740 cells per condition; *p* = 1; error bars are standard deviation of sample proportion and are small (∼0.1%). (B) Cytocompatibility images at different magnifications. Blue is all nuclei (Hoechst 33342); green is metabolically active cells (Calcein AM); and red is nuclei of dying cells (propidium iodide). (C) Alkaline phosphatase (ALP) expression, relative to that of hMSCs on matrices cultured in growth media (GM). *n* = 3 matrices; *p* = 0.22. (D) Whole-matrix and higher magnification images of fluorescently labeled ALP (red) and nuclei (blue). (E) Calcium deposit quantification using alizarin red S (ARS) labeling. *n* = 3 matrices; *p* = 0.007. (F) Color images of matrices labeled with ARS. (G) Relative gene expression of COL1A1 quantified from RT-qPCR. *n* = 3 samples from RNA pooled from cells cultured on three separate matrices per condition: *p* = 0.03. (H) Images of struts of 3DP-CaPG matrices. Blue is nuclei; red is ALP; gray is brightfield. (I, J) Compressive dynamic mechanical analysis determination of the (I) storage (*E*′) and (J) loss (*E*″) moduli of the matrices after 10 days of hMSC growth. *n* = 4 matrices; *p* = 0.21 and 0.17, respectively. Statistically significant differences are indicated by a line between data bars; unless otherwise indicated, error bars are standard error of the mean. Reproduced from ref (80). CC BY 4.0.

Using 3D printing, we were able to create porous constructs with tunable geometry that provide cellular access to the osteoconductive backbone while releasing key signaling molecules in a controlled manner. This approach aligns with the natural healing process, offering mechanical support and biochemical stimuli as the matrix degrades and new bone forms. Our 3DP-CaPG matrices have demonstrated osteogenic efficacy in both human mesenchymal stem cells (hMSCs) and in an orthotopic bone defect model, highlighting their potential as a clinically viable solution for bone regeneration (Figure 11). Despite these significant advancements, challenges remain in refining processing techniques for scalable production and patient-specific applications, which continues to be an area of active research.

#### 3.3.2 Hydrogels

GO is an important additive for hydrogels because it can serve as a bioactive and biocompatible filler, improving both the mechanical properties and bioresponse. Our group has done some work with GO in this capacity developing an injectable hydrogel for cartilage repair, integrating the natural regenerative capabilities of chondroitin sulfate (CS) with the mechanical strength and compatibility of a functional graphenic material derived from GO.^111^ We again used the Claisen functionalization method^20^ to create amine-functionalized graphene (EDAG) by intorducing primary amine functionality on the GO basal plane. This modification not only enhances nucleophilic reactivity for cross-linking but also improves the stability and compatibility when compared to GO. The carboxylic acids on CS were then activated using EDC/NHS chemistry allowing reaction with the primary amine of EDAG and forming EDAG–CS hydrogel. This injectable hydrogel can fill nonuniform defects and gel in situ without the need for external stimuli like UV light, making it ideal for minimally invasive cartilage repair. The anti-inflammatory properties of CS, combined with the enhanced mechanical strength and tunable interface of EDAG, create a robust, biodegradable scaffold that supports human mesenchymal stem cell growth and collagen production while preventing endochondral ossification.

Other groups have also recognized this potential. Both physical and covalent methods to incorporate GO into hydrogels have been investigated which were reported to significantly enhance the hydrogels’ properties for tissue engineering. Free-standing GO–gelatin methacrylate (GelMA) hybrid hydrogels were reported to have adjustable mechanical and electrical properties that are conducive to cellular functions, making them highly suitable as scaffolds.^121^ Dispersing GO and GelMA together in biological buffers, without the need for surfactants or sonication, yielded an uniform dispersion facilitated by strong noncovalent interactions. This technique not only boosts the mechanical properties of the hydrogels but also maintains essential cellular processes such as proliferation, migration, and morphogenesis. Moreover, NIH-3T3 fibroblasts encapsulated within GO–GelMA microgels exhibited similar spreading patterns and interconnected actin networks compared to pure GelMA, with an increase in proliferation, likely due to enhanced cell adhesion. These characteristics make GO–GelMA hydrogels highly desirable for creating robust, high-performing artificial tissues. In another work, GO was covalently integrated into hydrogels by chemically modifying GO with methacrylate groups (MeGO) and incorporating it via radical copolymerization.^122^ This approach enables the stable dispersion of higher concentrations of MeGO without aggregation, which improves fracture strength while preserving the rigidity of the hydrogels—an important factor for cell behavior.

##### 3.3.2.1 Conductive Hydrogels

While graphene oxide (GO) and reduced graphene oxide (rGO) derivatives offer tunability, they often exhibit higher electrical resistance due to the covalent attachment of charged functional groups to the basal plane of graphene.^22,123^ This increased resistance can diminish the material’s conductivity, making GO and rGO less suitable for certain electrical applications. The process of oxidizing graphite to GO provides a high-yield route to exfoliated carbon nanosheets that readily disperse in water due to hydrophilic oxygen groups on their surfaces. However, this oxidation compromises the graphitic nature of the nanosheets, leading to a loss of conductivity. Partial recovery of the graphitic character can be achieved through reduction by chemical, thermal, or electrochemical treatments, offering a degree of tunability based on the processing method. This tunability allows graphene to be optimized for specific applications, balancing its mechanical, electrical, and biological properties to suit the needs of desired biomaterial.

While conductivity of GO is reduced compared to graphene, the electrical conductivity of GO with higher C:O, or rGO ratios can be exploited in composites, creating elastic, conductive hydrogels suitable for cardiac tissue engineering and flexible electronics. GO incorporated hydrogels have been reported to simultaneously achieve both conductivity and elasticity, unlike alternatives such as gold nanowires or CNTs.^124^ For example, GO nanoparticles were covalently incorporated into conductive and elastomeric hydrogels of methacrylated tropoelastin, a key component of elastin.^125^ Incorporation of GO as additive to methacrylated tropoelastin enhanced both the elasticity and toughness of the hydrogels due to unique interactions between tropoelastin polymer chains and GO nanoparticles, including covalent bonds as well as hydrophobic, electrostatic, and hydrogen-bond interactions. The hybrid hydrogels are injectable, light-activated (due to methacrylation), and conductive (GO), making them suitable for applications like cardiac tissue engineering and flexible electronics. The incorporation of GO increased the elastic modulus and rupture strain, highlighting the improved mechanical properties. Additionally, these hydrogels demonstrated superior resilience to cyclic forces, supported cardiomyocyte growth and function, and showed biocompatibility with minimal inflammatory response, making them promising materials for tissue engineering and regenerative medicine.

The exceptional electrical and electrochemical properties of graphene and graphene oxide (GO) make them highly suitable for neural interface engineering. Their biocompatibility, coupled with their ability to promote neural cell adhesion and growth, gives them a significant advantage over other materials. Graphene has been found to exhibit electrochemical capabilities like gold and platinum for neural recording. rGO-based flexible neural recording electrodes have been developed as three-dimensional flexible membranes for long-term recordings and two-dimensional thin films for acute recordings.^126^ Both types of electrodes showed a stable recording for up to 21 days in chronic in vivo experiments. 3D flexible membranes provided a porous interface leading to better penetration of neurons and long-term recording.^126^ rGO-based 2D electrodes on the other hand offer a nonporous interface with better electrical properties and no penetration of neurons, and thus were more suitable for acute recordings and easy removal.^126^

Graphene-based flexible membranes have emerged as promising tools for neurostimulation and neural signal recording. Traditional neural stimulation systems often rely on sharp metal microelectrodes, which exhibit poor electrochemical properties, causing significant tissue damage and compromising the long-term stability of implantable devices. In contrast, graphene is being explored as a superior alternative for designing flexible cortical microelectrode arrays.^127^ A notable approach involves patterning porous graphene spots onto a polyimide film using direct laser pyrolysis.^127^ These porous graphene-based flexible microelectrodes enable efficient electrophysiological sensing and stimulation from the brain surface without penetrating the tissue, offering a less invasive and more biocompatible solution.^127^

Graphene-based microtransistors have been shown to effectively record infraslow brain activity through in vivo recordings.^128^ In these studies, graphene solution-gated field-effect transistors (gSGFETs) were utilized for both epicortical and intracortical mapping of cortical spreading depression in rats. These graphene microtransistors demonstrated comparable performance to solution-filled glass micropipettes while also enabling spatially resolved mapping. Furthermore, gSGFETs were successfully integrated with optical techniques, such as laser speckle contrast imaging (LSCI), to generate 2D maps of neurovascular coupling.^128^

Notably, INBRAIN Neuroelectronics, a pioneer in brain–computer interface (BCI) therapies, achieved a groundbreaking milestone by implanting its graphene-based cortical interface in a human patient.^129^ This first-ever human application of a graphene-based BCI underscores the transformative potential of graphene-based neural technologies in medicine.

### 3.4 FGMs and GO as Agents for Controlled Delivery

Graphene has gained significant attention for its potential in advanced drug delivery systems. However, pure graphene faces challenges such as cytotoxicity, poor solubility in physiological environments, and toxicity risks. The biocompatibility of graphene-based nanomaterials depends on several factors, including size, surface charge, shape, layer number, and surface functional groups. While some studies have shown no toxicity in mouse cells, others reported considerable toxicity, such as membrane damage in Hep-G2 cells and DNA fragmentation in human mesenchymal stem cells.^130,131^ Aggregated graphene particles can also induce oxidative stress by increasing reactive oxygen species (ROS), leading to protein, DNA, and lipid damage.^35,132^ FGMs offer enhanced water solubility and biocompatibility, making them more suitable for biomedical applications. Functionalizing GO, covalently or noncovalently, allows the incorporation of active drugs or biomolecules. Specifically, hydrophilic groups, such as hydroxyl, epoxide, and carboxyl, not only enable good dispersion in physiological environments, but can also enhance drug delivery potential.

#### 3.4.1 Delivery of Small Molecules and Ions

Even before 2010, GO was recognized as a potentially carrier for hydrophobic anticancer drugs.^133^ Many potent hydrophobic drugs, particularly aromatic compounds, face challenges in clinical use due to their poor water solubility. In this 2008 study, PEGylated nanographene oxide (NGO-PEG) was used to noncovalently complex with SN38, a camptothecin (CPT) analogue, via van der Waals interactions. The resulting GO–drug complex exhibited excellent water solubility, retaining the potency of SN38, which is significantly more toxic than its FDA-approved prodrug, irinotecan (CPT-11). Here, GO was synthesized using a modified Hummer’s method and, while soluble in water, it aggregated in salt-rich or protein-containing solutions. This aggregation was attributed to the screening of electrostatic charges and nonspecific protein binding. The resulting PEGylated NGO demonstrated excellent stability in biological media, including serum and sustained release, with only about 30% release in serum over 3 days. This approach proved generalizable, as other hydrophobic, aromatic drugs, including CPT analogues and the EGFR inhibitor gefitinib (Iressa), were also successfully loaded onto NGO-PEG through simple adsorption.

In a similar strategy, GO was covalently bound to chitosan via an amide bond, resulting in functionalized graphene oxide chitosan (FGOCs).^134^ The covalent modification reduced GO, leading to stable dispersions that lasted several days to months. The negative charges of terminal carboxylic acid and protonated amine groups in chitosan contributed to the stability through electrostatic repulsion. The study examined the controlled release of two drugs, ibuprofen (IBU) and 5-fluorouracil (5-FU), from FGOCs. The results suggest the potential of FGOCs as targeted drug delivery systems that enhance the efficacy of chemotherapeutics while minimizing toxicity to nontarget cells. A similar approach has been widely explored, where GO is functionalized with natural or synthetic polymers, allowing for the physical adsorption of drugs through noncovalent interactions. This method has enabled GO-based drug delivery platforms for a wide range of therapeutics, including anticancer, antibacterial, antibiotic, antiemetic, antimicrobial, antidiabetic, antituberculosis drugs, NSAIDs, flavonoids, proteins, and antimigraine medications.

FGMs can also be used to deliver inducerons, which are ions that induce bioactivity.^78^ Using water-labile covalent bonds, FGMs can be synthesized to deliver sustained release of inducerons, creating intrinsically bioactive scaffolds. For example, we synthesized phosphate graphenes (detailed in Section 3.1.1) capable of delivery of Ca^2+^, K^+^, Li^+^, Mg^2+^, Na^+^, and PO_4_^3–^ and found that the material that delivered Ca^2+^ and PO_4_^3–^ to be intrinsically osteoinductive to MSCs, without the use of growth factors, in vitro and in vivo.

#### 3.4.2 Delivery of Peptides

Efforts have been made to use GO-based systems for peptide delivery, either through covalent or noncovalent interactions. A general approach to load peptides onto GO sheets has been electrostatic interactions between GO and peptides. However, since GO is negatively charged, covalent conjugation of positively charged polymers is performed to improve the electrostatic interaction ability of GO with the negatively charged peptides.^135^ Another commonly used approach is covalent conjugation of a bioactive peptide, either directly to GO or through use of a spacer.^91^ For example, as spacer was used to conjugate human neuropeptide Y (NPY), a modulator of neuronal transmission, to GO.^136^ GO was functionalized with 11-azido-3,6,9-trioxaundecan-1-amine (TEG-N_3_) via epoxide ring opening, producing GOTEG-N_3,_ followed by a copper-catalyzed azide–alkyne click reaction to link the NPY peptide. Notably, the conjugation process did not affect NPY’s α-helical conformation, which could be a benefit of using the spacer. NPY bound to GO maintained its biological activity with an extended effect compared to free NPY, suggesting enhanced pharmacokinetics.

Another similar study explored two different methods for coupling the antimicrobial peptide Chicken cathelicidins (CATH-2) with reduced graphene oxide (rGO): covalent and noncovalent interactions.^137^ Covalent bonding was achieved using biocompatible cross-linkers (EDC/NHS), while noncovalent interactions relied on a self-assembly approach via electrostatic or π interactions. The covalently conjugated peptide–rGO complex showed enhanced antibacterial activity against *Escherichia coli* with lower cytotoxicity toward erythrocytes compared to the self-assembled complex or rGO alone. The antibacterial mechanism likely involves membrane stress, leading to degradation, loss of integrity, and cell lysis.

Supramolecular hydrogels show promise for drug delivery due to their injectability and precision in targeting. However, their weak mechanical stability and rapid erosion often limit their effectiveness in vivo. Traditional hydrogels release drugs via diffusion or decomposition, making controlled, on-demand release challenging. To address these limitations, a novel peptide–GO hybrid hydrogel was designed offering enhanced stability, injectability, and NIR-triggered drug release for precise dosage control.^138^ The design incorporates a peptide with a pyrene motif for binding to GO, a glycine-alanine (GA) sequence for β-sheet formation, and a tyrosine residue for photo-cross-linking. These peptides bind noncovalently to GO through π–π interactions, while GA β-sheets facilitate cross-linking between GO layers. Photo-cross-linking of tyrosine residues adds further stability. The system is designed with both strong covalent bonds (dityrosine) for structural integrity and weaker hydrophobic and hydrogen bonds for responsiveness to external stimuli. The robust structure ensures the hydrogel is suitable for triggered drug release, offering advantages over conventional GOS-drug hybrid hydrogels, which often lack mechanical strength and decompose prematurely during drug release.

### 3.5 Future Directions

Over the past decade, there has been a significant body of work focused on graphene oxide (GO), yet some researchers may observe a slight decline in the number of publications on the topic. A Google Scholar search for the keyword “Graphene oxide” shows a 56% decrease in publications from 2020 to 2023. However, I do not believe this signals a diminished interest in GO for biomedical applications. In fact, when examining the keyword “Graphene oxide biomaterial”, there has been a notable 38% increase in publications during the same period. This suggests that rather than moving away from GO, researchers have identified its unique chemistries and properties as ideally suited for emerging applications in human health (Figure 10).

## 4 Emerging Technologies in Public Health

The prevalence of work over the past decade suggests the biocompatibility and degradability of GO, making it suitable as a biomacromolecule to be used in a variety of biomedical applications (Figure 10). However, as researchers determined these compatibility features, it can also be noted that if a material is biocompatible, it is likely also eco-compatible. Similar work to that done in vitro and in vivo must verify that GO is compatible and nontoxic in the environment, both freshly prepared and as it degrades, and this work is beginning to emerge.^61,139^ However, this work builds on the foundation of published biocompatibility work, and there is strong promise for GO-based biomaterials for applications in sustainability and public health.

In coming years, a larger importance will be placed on public health areas.^140^ With the world population expected to exceed 9 billion by 2050,^141^ new socioeconomic pressures are being placed, with biomaterials research poised to provide materials solutions to these problems. Growing pains force a larger discrepancy between rich and poor countries in aspects of life from healthcare to food quality. There is a society responsibility to seek out cost-effective solutions to improve safety and health universally.

It is my belief that biomacromolecules stand poised to address these issues. Biomimetic FGMs have been used to purify drinking^142^ and wastewater,^143−145^ and with responsible design and implementation, these materials can be designed and employed to prevent further exposure incidents like the Flint Michigan Water crisis.^146,147^ However, as we develop these new materials, it is also important to develop methods to consider the lifecycle of the material to ensure long-term safety.

### 4.1 Environmental Toxicity

Before creating a material of the future, especially for sustainability and public health applications, the toxicity of the material and its degradation pathways must be considered. This generation of chemist is only now realizing the dangers of the last generations’ “miracle materials”. For example, issues with materials such as asbestos, disposable plastics, and per- and polyfluoroalkyl substances (PFAS) were only realized decades after widespread use and have become one of the largest challenges for materials researchers today. Considering this, our generation of researcher must be more prudent, and materials must be developed in light of the entire material lifecycle.

The commercial production and use of FGMs are rapidly increasing, with projections suggesting further growth. Their ability to interact with diverse materials—such as drugs, polymers, and metals—has led to widespread applications in electronics, composites, drug delivery, tissue regeneration, and environmental remediation. However, this extensive use raises concerns about environmental exposure, particularly in soil and water. GO-polymer nanocomposites, for instance, may release GO particles due to degradation, and FGM-containing products can introduce these materials into ecosystems during disposal. Extensive use of FGMs in biomaterials is another potential avenue through which FGMs or their biodegradation products such humic acid can be exposed to the environment. While FGMs are considered biocompatible,^63,148^ their long-term ecological effects remain largely unknown, echoing the concerns seen with substances like PFAS. Unregulated PFAS use has led to widespread environmental contamination and human health issues due to their persistence and bioaccumulation. To prevent a similar scenario, it is essential to study the environmental impact of FGMs early on, ensuring that their growing use does not lead to unintended consequences. It is also crucial to develop methods to assess the impact of these graphenic materials on the environment in a high-throughput manner and being able to make estimations about the feasibility of their long-term use.

#### 4.1.1 Interactions of GO and FGMs with the Environment

FGMs upon exposure to environment are expected to interact with air, soil and water being the three most important systems. The FGMs are thus expected to undergo transformation upon interaction with a variety of environmental media such as dissolved organic matter, ions, UV, microorganisms and biota. This interaction will lead to a range of physical, chemical and biological transformations further impacting the environmental toxicity of these materials.

##### 4.1.1.1 Interaction of FGMs with Air

Graphene-based materials released into the air can remain suspended for extended periods, with high mobility and strong adsorption, posing potential biological risks. These materials tend to agglomerate due to van der Waals forces and π–π stacking, which depend on their structure. Their strong adsorption capacity might also lead to interactions with airborne particles, ozone, and gases, altering their size, charge, or functional groups. Studies show that ozone can change graphene’s surface morphology and hydrophilicity through physical adsorption, while sunlight can transform or degrade graphene oxide. However, most studies focus on GO in suspension, leaving knowledge gaps about its behavior in the air.

##### 4.1.1.2 Interaction of FGMs with Soil

Soil is a critical environment where FGMs can migrate and undergo transformation.^149^ Recent studies have highlighted the challenges of GO mineralization in soil, revealing that GO is resistant to conversion into CO_2_ and is not easily released by water due to its tendency to aggregate with soil colloids, forming larger particles that remain in the soil.^150^ Research has also focused on how factors like ionic strength (IS) and pH influence GO’s transport and retention. For example, it was observed that GO displayed higher mobility in low IS conditions (1 mM NaCl) and that retention significantly increased with higher IS. Over time, GO breakthrough concentrations increased across all IS levels as deposition sites became saturated, reducing deposition rates. Changes in IS also alter the surface properties of sand and GO, such as zeta potential, leading to suppression of the electric double layer.^151^ Furthermore, it was found that inhibition of GO transport depends on the hydrated radius, following a trend of Na^+^ < K^+^ < Cs^+^ and Mg^2+^ < Ca^2+^ < Ba^2+^.^152^ Soil composition, including the presence of organic matter and clay minerals, also impacts GO transport. rGO transport, for instance, was enhanced in the presence of humic acid due to adsorption on the GO surface.^152^ Soil moisture, texture, and porosity further affect the deposition of FGMs. One study simulating soil columns showed that as soil porosity decreased, the leaching of rGO-Pd nanosheets initially increased and then decreased. This process also altered the physical and chemical properties of rGO-Pd, including its morphology, thickness, and oxygen-containing surface groups.^153^

Microorganisms in the soil play a vital role in the degradation and transformation of graphene-based materials. Nitrogen-fixing bacteria have been reported to reduce GO in the soil, increasing its toxicity.^154^ Fungi, particularly white rot fungi like *Phanerochaetes chrysosporium*, have been reported to efficiently decompose FGMs by adding oxygen to rGO, increasing defects in its carbon skeleton and exfoliating graphene sheets.^155^ Studies on *Bjerkandera adusta*, *P. chrysosporium*, and *Morchella esculenta* showed that while no structural changes occurred in few-layer graphene, oxidation to graphene-like substances was evident over time. Longer incubation may further increase oxidation, resulting in lattice pores and fragmentation.^156^ Certain plants, such as crops, also interact with GO. In agriculture, GO has been shown to accumulate in rice, enhancing the accumulation of polycyclic aromatic hydrocarbons when coexposed.^157^ GO can transfer through the food chain, causing plant interactions with other pollutants. In addition, FGMs undergo redox reactions in soils rich in sulfides or ferrous ions and can immobilize heavy metals like chromium through electrostatic interactions and surface complexation.^158^ Overall, the transformation of FGMs in soil is influenced by their intrinsic properties, solution chemistry, soil characteristics, and microbial activity, leading to processes such as aggregation, deposition, adsorption, degradation, and redox reactions.^149^

##### 4.1.1.3 Interaction of FGMs with Soil Organisms and Plants

A decade of research in biomaterials suggests the potential environmental compatibility of GO and FGMs, however, due diligence must be done to prove its safety in these environments. To address this, my group has been working to create assays to evaluate the environmental safety of these materials using *Caenorhabditis elegans* (*C. elegans*). In addition to the safety of these materials as-prepared, it is critical to also understand the effects of the material on the environment as the material degrades, as well as develop methods to simulate this aging effect to match longer time scales found in nature.

**Figure 12:**
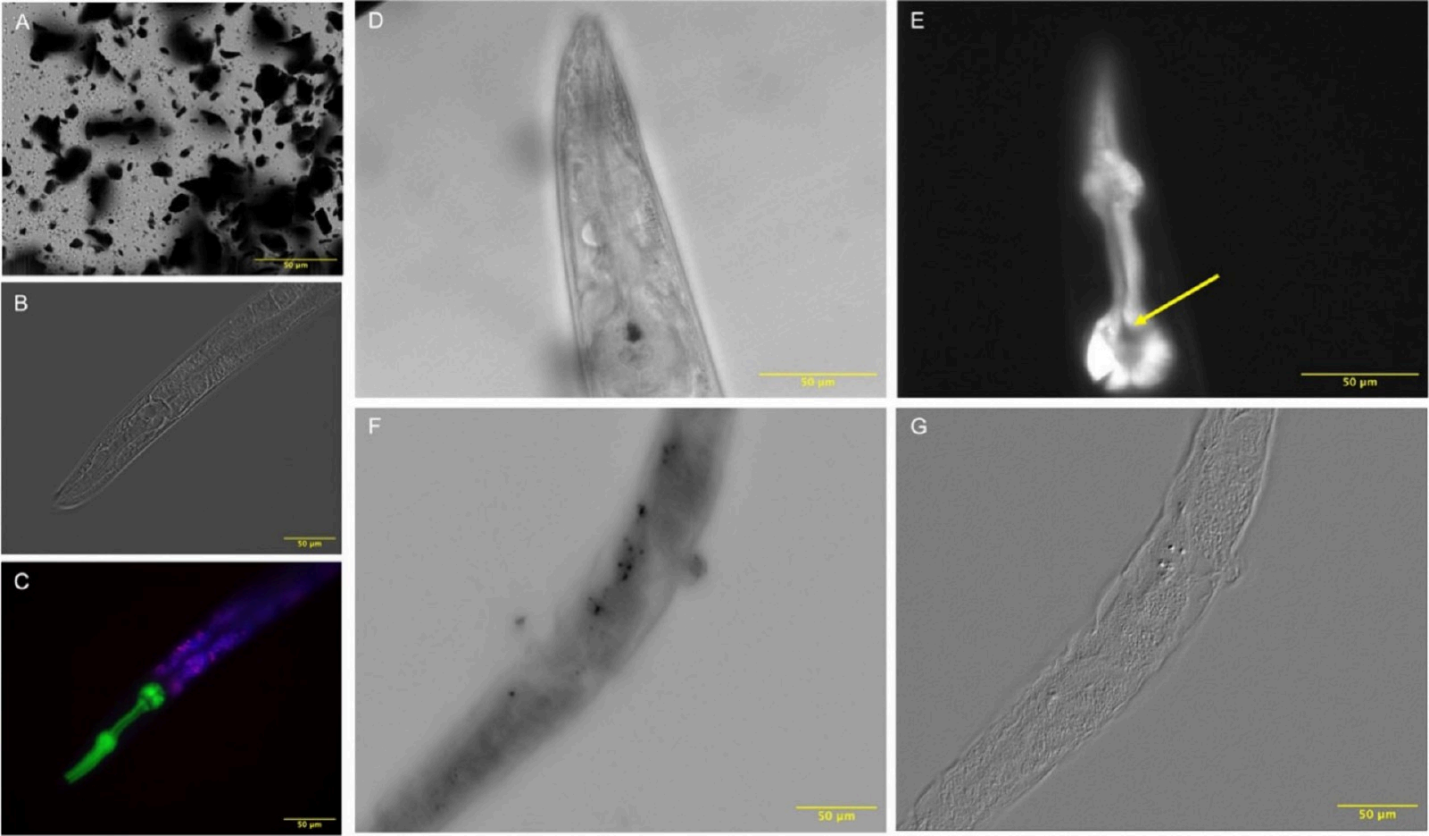
Transmitted light and fluorescence microscopy of CGC91:*C. elegans* after chronic exposure to GO. All scale bars measure 50 μm. (A) A high-concentration GO solution (40×) in transmitted light shows a wide variety of particle sizes for *C. elegans* to interact with. (B, C) Control images were taken of untreated *C. elegans* in transmitted light and fluorescence, respectively (25×). (D, E) Transmitted light and fluorescence images showing GO in the pharynx of a nematode that was chronically exposed to GO (40×). In (E), the arrow indicates the location of the nonfluorescent particle. (F) Average axial projection of transmitted light images from a GO-exposed nematode showing dark particles within the body of the worm (25×). (G) Difference-contrast transmitted light image corresponding to (F) highlighting the location of the in-focus particles relative to the internal features of the worm (25×). The images confirm that *C. elegans* ingests FGM particles. GO particles showed only very weak luminescence in the fluorescence microscope under these experimental conditions. Reproduced with permission from ref (61). Copyright 2023 Elsevier.

To this end, our group recently published a study toward the environmental impact of FGMs by assessing their toxicity on model organisms found in soil and aquatic environments—bacteria and nematodes.^61^ A range of FGMs were selected, varying in chemical properties such as oxidation level, size, charge, and ultrasonic alteration. GO and rGO were tested, along with other modified FGMs like CG and polylysine-grafted CG (pKCG), to simulate common modifications in graphene-based products. Ultrasonication was used to accelerate material breakdown in aqueous conditions, mimicking the environmental degradation of FGMs. The study focused on the impact of these graphenic materials in fresh and altered states on bacterial viability (*E. coli* and *Bacillus subtilis*) and the behavior of *Caenorhabditis elegans*, a nematode commonly used as a model for soil and water exposure. The results showed that FGMs had minimal impact on both bacteria and nematodes. Bacterial viability remained comparable to control groups, regardless of FGM oxidation levels, size, or surface charge. Similarly, *C. elegans* exhibited no significant changes in fertility, locomotion, or general health after chronic exposure to the FGMs, including the ultrasonically altered versions. Ingestion of FGMs was observed in the nematodes, but there was no evidence of toxicity or adverse effects, regardless of particle size (Figure 12). These findings suggest that FGMs can be designed to minimize environmental impact and toxicity, but further research is needed to fully understand their long-term ecological effects. The work also established a robust and scalable environmental screening system for assessing two-dimensional nanomaterial toxicity.

Presence of FGMs in soil could impact the health of plants, as well as soil-based organisms (such as nematodes). Moreover, the possibility of these graphenic materials leaching back into the plant leaf, fruits could bring them back into the food cycle. It is important to consider this effect as well, and studies are underway. For example one of the early studies evaluated the impact of GO on plant growth and physiology using cabbage, tomato, red spinach, and lettuce, with graphene concentrations ranging from 500 to 2000 mg/L.^159^ After 20 days of exposure, graphene significantly inhibited root and shoot growth, reduced biomass, and decreased the number and size of leaves in a dose-dependent manner compared to the control. In cabbage, tomato, and red spinach, the exposure led to a concentration-dependent increase in reactive oxygen species (ROS) and cell death, with visible symptoms such as necrotic lesions, indicating oxidative stress-induced damage. However, lettuce seedlings showed little to no toxic effects under the same conditions. The results suggested that the toxicity of graphene is highly dependent on dose, exposure duration, and plant species. The overproduction of ROS appears to play a critical role in graphene-induced toxicity, leading to inhibited plant growth and reduced biomass. Signs of necrotic damage, electrolyte leakage, and H_2_O_2_ accumulation further support an oxidative stress mechanism. These findings highlight the need for extended studies on the toxicity of graphene and its potential long-term risks to terrestrial plants under varying concentrations and exposure times.

##### 4.1.1.4 Interaction of FGMs with Aquatic Environments

The leaching of GO into aquatic environments has raised concerns about its potential impact on both the balance of essential nutrients and the health of aquatic organisms. Once in the water, GO can interact with planktonic and benthic crustaceans,^160^ such as *Daphnia magna*(161) and *Thamnocephalus platyurus*,^162^ through contact with their bodies, skin, and gills or through ingestion. These interactions can affect the organisms’ physiology and behavior, with crustaceans showing varying sensitivities to GO toxicity based on their feeding strategies. GO also poses risks to primary producers like algae and cyanobacteria, which are crucial for oxygen production, nutrient cycling, and biomass generation in aquatic ecosystems. Studies have shown that cyanobacteria, due to GO’s antibacterial properties, are more sensitive to GO toxicity compared to algae. GO exerts harmful effects through multiple mechanisms, including shading, nutrient adsorption, and a physical “nanoblade” effect that damages cell membranes. While these mechanisms cause initial toxicity, algae and cyanobacteria can mitigate these effects over time by activating resistance mechanisms, such as the production of extracellular proteins and carbohydrates.^160^

Like in mammalian systems, the degree of GO oxidation also plays a significant role in its ecotoxicity. Highly oxidized forms of GO generate increased oxidative stress, but organisms are able to counteract this through antioxidant enzyme activity, reducing overall toxicity. Less oxidized on the other hand, causes mechanical damage to aquatic organisms. However, studies have shown that pretreatment of crustaceans with algae can significantly alleviate GO’s toxic effects, reflecting conditions in natural ecosystems.^162^ This suggests that the environmental risk of GO may be lower than previously thought, as the presence of food sources like algae can mitigate both mechanical and oxidative damage. Overall, these findings emphasize the importance of considering organism-environment interactions, such as the availability of food, when assessing the potential environmental impact of graphene oxide in aquatic ecosystems.

In aquatic environments, multiple adsorbates often coexist and can coadsorb onto FGMs. Similar to that of soil, factors such as pH, presence of organic molecules and inorganic ions, ionic strength can impact the transformation of FGMs in the aquatic environment. Inorganic ions can shield the surface charge of FGMs, enhancing the adsorption of negatively charged^163^ organic molecules while suppressing positively charged^164^ ones. Increased ionic strength can also promote a “salting-out” effect, reducing the solubility of organic molecules and altering their adsorption. This effect is more pronounced for ionized molecules than for nonionized ones.^165^ Metal ions tend to adsorb more readily onto GO than rGO due to its abundant surface functional groups, with solution pH being a dominant factor in metal adsorption.^166^ Natural organic matter adsorbs onto GO via hydrogen bonding, Lewis acid–base interactions, and π–π interactions.^167^ FGMs can also adsorb various organic molecules (e.g., amino acids, proteins) and inorganic ions. An early study highlighted the depletion of essential micronutrients such as folic acid and pyridoxine in human cells exposed to graphene, suggesting a potential starvation toxicity mechanism.^168^ This nutrient depletion may occur in aquatic organisms as well, although it has not been reported. Additionally, FGMs’ high adsorption capacity can interfere with in vitro toxicology assays by preventing molecular probes from reaching their targets, quenching fluorescent signals, or altering probe redox states.

### 4.2 Use of FGMs and GO in Water Purification

The unique structure of GO, characterized by both sp^2^ (aromatic) and sp^3^ (aliphatic) carbons alongside oxygen-containing functional groups, opens a wide array of possibilities for its use in water purification systems. The functional groups allow for direct interactions with metal and small molecule impurities. The polar functional groups not only make GO highly hydrophilic but also enable its stable dispersion in aqueous solutions, which is essential for membrane fabrication. Furthermore, the low cost of GO make it an attractive material for researchers looking to develop separation processes. By controlling parameters such as oxidation levels, size, and the functional groups present during synthesis, the lamellar arrangement of GO can be optimized to significantly enhance separation efficiency.

FGMs have emerged as a promising candidate for the fabrication of highly selective and permeable membranes, especially in water purification. What sets graphene-based membranes apart from traditional inorganic or polymer-based membranes is their ability to combine high permeability with selectivity, often outperforming conventional options. However, much of this success comes from creating extremely thin membranes, sometimes less than 50 nm, which is a key factor in achieving high permeation flux.^142,169,170^ To further enhance performance, graphene-based membranes are frequently modified with nanomaterials, ranging from inorganic nanoparticles to organic molecules. These modifications enable a trade-off between permeability and selectivity by improving the surface properties of the membranes. Nanomaterials, particularly those with polar functional groups, facilitate the creation of frictionless capillary channels, which enhance the speed of fluid transport through the membrane, thus boosting overall permeability.^171,172^

#### 4.2.1 Design of GO Filtration Membranes

The performance of membranes in contaminant removal is not only influenced by the composition and intrinsic properties of the materials, but also by nanoarchitecture. The integration of nanomaterials into membranes has been a focal point of research, particularly for applications in extreme environments or nonaqueous solvents. Compared to traditional polymeric and ceramic membranes, graphene-based membranes offer several advantages, including high solvent resistance, thermal and chemical stability, and superior mechanical strength.^173^ These characteristics have revolutionized the field of nanofiltration, enabling graphene-based materials to efficiently remove a broad spectrum of contaminants, such as organic pollutants, heavy metals, pathogens, and pharmaceuticals. Moreover, these membranes exhibit high molecular selectivity across a range of solvents, including harsh organic environments, which adds to their versatility and appeal. Like its use in traditional biomaterials, consideration must be given to processing, as use of a powder material in application is not obvious. Thus, in fabricating graphene-based membranes, various methods such as layer-by-layer assembly, spin coating, dip coating, and vacuum-assisted deposition play pivotal roles.^173^

##### 4.2.1.1 Vacuum Filtration Method

In this approach vacuum filtration of GO dispersion is performed to yield GO membranes deposited on traditional ultrafiltration or microfiltration base membranes. The hydrophilic and negatively charged functional groups (epoxy, hydroxyl, carboxyl) on GO enable its stable dispersion in water, facilitating the vacuum or pressure-assisted fabrication of multilayered GO membranes. In these filtration techniques, GO nanosheets are deposited in parallel on ultrafiltration or microfiltration base membranes. By adjusting the volume or concentration of the GO solution, membrane thickness can be controlled. Studies show that pressure-assisted methods produce GO membranes with highly controlled laminar structures and minimal surface roughness. Micrometer-thick GO laminates formed via vacuum filtration were found to be impermeable to gases, but when immersed in water, they acted as molecular sieves, blocking solutes with hydrated radii larger than 4.5 Å.^174^ Smaller ions passed through at rates significantly higher than expected for simple diffusion. This behavior was attributed to the formation of nanocapillaries in the hydrated state, allowing only appropriately sized species to pass. The fast ion permeation was attributed to a capillary-like pressure effect inside the graphene capillaries. The vacuum filtration method is a simple and facile way to create filtration membranes from an aqueous solution; however, the obtained ultrathin membranes are easily redispersed into water during water-based operations. As a result, it is difficult to control the interlayer distance and reduce the selective layer thickness of the membranes for practical industrial applications.^175^

##### 4.2.1.2 Spin Coating/Dip Coating Method

Techniques like spin coating leverage centrifugal shear forces to evenly distribute GO across substrates, creating ultrathin membranes.^173^ Dip coating involves immersing substrates into GO solutions and slowly withdrawing them, allowing a GO layer to form as the solvent evaporates. Drop casting, another prevalent method, entails placing small droplets of GO solution onto a substrate and allowing them to dry. These methods are particularly beneficial for designing freestanding graphene oxide structures suitable for diverse filtration and separation applications. For instance mechanically robust, chlorine-tolerant GO composite membranes were designed through a spin-coating method that integrates GO with an organic monomer solution (monofunctional *N*-isopropylacrylamide and difunctional *N*,*N*′-methylenebis(acrylamide) as monomers, and ammonium persulfate as initiator) onto a porous polymer substrate, followed by polymerization.^175^ This resulted in an ultrathin, highly cross-linked GO-polymer layer, less than 40 nm thick, which offers significant advantages in forward osmosis desalination, providing high water flux and excellent salt rejection. Specifically, the membranes demonstrated a NaCl rejection rate of 99.9%, underscoring their potential in robust and efficient desalination applications. These findings illustrate the versatility of graphene-based membranes, particularly in terms of mechanical stability and performance efficiency in harsh environments.

##### 4.2.1.3 Layer-by-Layer Self-Assembly

Another common method for creating GO based filtration membranes is layer-by-layer (LBL) self-assembly method which creates a molecularly charged thin film by layering polyelectrolytes of opposite charges. These LBL membranes, composed of polycations and polyanions, form through electrostatic interactions over a charged substrate.^173^ This technique works well with GO owing to its laminar structure and charged oxygen functional groups. Using this technique, a high-performance composite membrane was designed by depositing a GO nanofiltration layer onto hollow fiber supports for heavy metal removal.^176^ Torlon 4000T-MV polyamide-imide was selected as the substrate due to its mechanical and thermal stability. The surface of the substrate was modified with hyperbranched polyethylenimine, introducing free amine groups for GO attachment. A layer-by-layer deposition of GO nanosheets was applied, followed by cross-linking with ethylenediamine for enhanced stability. After 5–10 deposition cycles, surface defects were sealed, reducing pore size and narrowing pore size distribution. These GO composite hollow fiber membranes demonstrated excellent water permeability and efficient rejection of heavy metals like Pb^2+^, Zn^2+^, and Ni^2+^. The membranes also showed long-term stability in a 150 h rejection test for Pb^2+^.

#### 4.2.2 Dispersive and Column Purification

**Figure 13:**
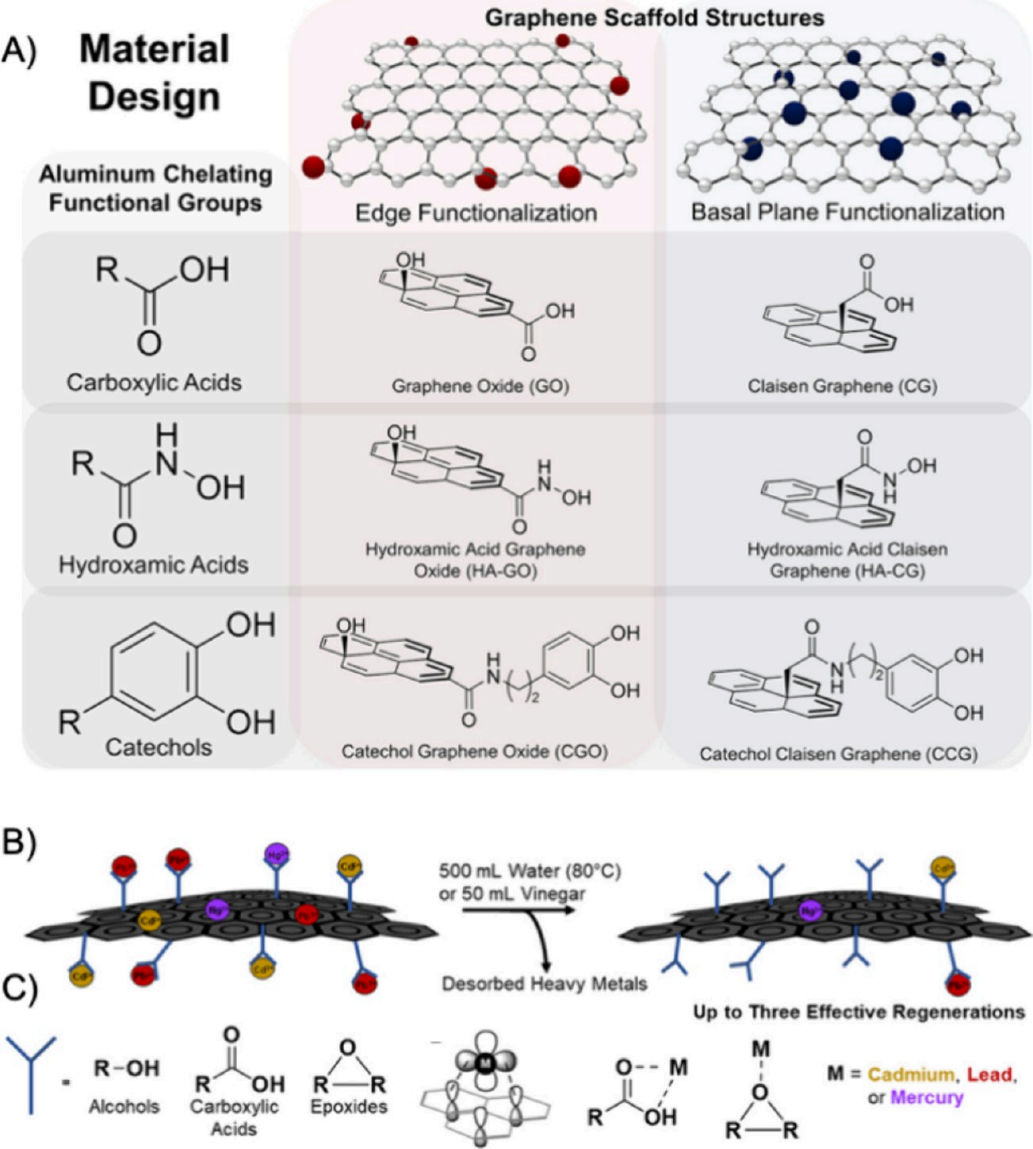
(A) Material design of aluminum-chelating functional graphenic materials (FGMs) for acid mine drainage remediation. Aluminum-chelating functional groups (carboxylic acids, hydroxamic acids, and catechols) are represented on the left and the corresponding FGM scaffold containing the functional group is contained within the same row. Edge-functionalized FGMs are represented in red and basal-plane-functionalized FGMs are represented in blue.^145^ (B) Adsorption and regeneration illustration of cadmium, lead, and mercury with functionalized graphene-based regeneratable filters through alcohols, carboxylic acids, and epoxides. (C) Functionalized graphene can adsorb metals through d−π conjugation or polar adsorption via oxidized functional groups.^142^ Reproduced from ref (142). CC BY 4.0.

Our group first recognized the potential of FGMs in water purification for wastewater applications. In the counties surrounding Carnegie Mellon and Pittsburgh, acid mine drainage presents a significant environmental threat. Acid mine drainage is typically acidic, dissolving both iron and aluminum, so conventional remediation efforts rely on pH buffering to precipitate these metals. Specifically, the challenge lies in the fact that iron can be precipitated out under acidic conditions, whereas aluminum requires much higher pH values to precipitate, allowing it to persist in the environment. To address this, we designed a FGM-based scrubber to remove persistent aluminum via chelation specific for dissolved aluminum (Figure 13A).^145^ We found that these FGM-based scrubbers could remove aluminum from acidic water, chelating up to 21 μg Al/mg of FGM. Because of our extensive biomedical studies on FGM compatibility, we were not surprised to find these materials to be environmentally nontoxic, based on preliminary tests. Interestingly, however, we also found that the oxygen-based functional groups intrinsic to GO and CG—epoxides, carboxylic acids, and alcohols—were effective, nonspecific metal chelators, enabling their use in filtration devices without further chelation functionalization.

#### 4.2.3 Regeneratable GO/CG Filtration Membranes

Using this type of filtration device, my group recently created a graphene-based water filter that can be regenerated at home using either hot water (80 °C) or vinegar, for up to three regeneration cycles.^142^ This innovation offers a cost-effective and environmentally friendly alternative to frequent replacement of expensive filters. Lead adsorption experiments showed GO is more efficient at lead removal, while CG regenerates better due to its higher carboxylic acid content, which responds dynamically to pH changes. After the first regeneration, metal adsorption of CG increased by 34.9%, likely due to the protonation and deprotonation of its carboxylic acids in response to pH variations, especially at elevated temperatures. The 90:10 GO/CG filter demonstrated strong removal capabilities for lead, cadmium, and mercury, metals of concern to the FDA (Figure 13B,C). Breakthrough curves showed that lead adsorption took the most volume to reach saturation, which is particularly relevant for addressing environmental health concerns related to lead contamination in domestic water supplies.

#### 4.2.4 Promise of GO-Based Filters in PFAS Removal

GO and FGMs are also extremely promising materials for removal of “forever chemicals” PFAS now present in wastewater.^177^ Surfactant-modified GO has shown improved dispersibility and stability in both aqueous and organic solvents, with the interaction between surfactants and GO depending largely on surfactant concentration and characteristics. Researchers have explored the potential of surfactant-enhanced GO for wastewater treatment. For example, hexadecyltrimethylammonium bromide, a cationic surfactant can be employed to modify a GO-based adsorbent, achieving nearly 100% removal of copper ions and bisphenol A within 1–2 h.^178^ In fact, the surfactant/GO composite demonstrated 15 times the adsorption capacity of unmodified GO. Researchers have also started exploring surfactant-modified GO for removing PFAS from water. A recent study aimed to fill this gap by comparing the adsorption performance of seven surfactant-modified GOs for removing 11 PFAS compounds.^179^ Among these, GO modified with the cationic surfactant cetyltrimethylammonium chloride (CTAC) stood out. The GO-CTAC composite exhibited a highly positive surface charge, significantly enhancing its adsorption efficiency. In pure water, GO-CTAC removed nearly 100% of the target PFAS within 1 h, and its effectiveness remained high regardless of changes in pH, NOM concentration, or ionic strength. In river water samples, it achieved nearly complete removal of 10 PFAS in 4 h. The results suggest that electrostatic and hydrophobic interactions play key roles in the adsorption process, positioning GO-CTAC as a fast-acting and resilient adsorbent for PFAS removal across various water environments. This study adds to the growing body of research seeking effective materials for addressing PFAS contamination.

Another study by Foudazi and co-workers reported the design of a GO-based nanocomposite adsorbent called magnetic amine-functionalized graphene oxide (MAGO).^180^ This was achieved by covalently grafting amine groups onto GO surfaces and incorporating iron oxide (Fe_3_O_4_) nanoparticles for magnetism. MAGO was evaluated for its adsorptive removal of both long-chain and short-chain PFAS, including perfluorooctanoic acid, perfluorooctanesulfonate, perfluorohexanesulfonate, and perfluorobutanesulfonate. The results were striking—MAGO achieved over 95% removal for long-chain PFAS and over 85% for short-chain PFAS in just 30 min, showcasing rapid adsorption kinetics. Moreover, it maintained performance across a broad pH range (4 to 7), indicating robust stability in diverse water conditions. The high removal efficiency was attributed to the synergistic interactions between GO and the amine functional groups, which promote both electrostatic attraction and hydrophobic interactions with PFAS molecules. This dual-mode interaction enhances adsorption, especially for long-chain PFAS. Additionally, MAGO demonstrates significant regeneration capabilities, making it a highly effective and sustainable option for PFAS removal. These further underscores the versatility of graphene-based adsorbents in environmental remediation, particularly in addressing complex contaminants like PFAS.

Overall, the ability to manipulate the structure, composition, and fabrication techniques of graphene-based membranes has opened new avenues for improving separation performance. By fine-tuning factors like membrane thickness, surface roughness, and functional group composition, it is possible to create membranes that not only meet the demands of specific applications but also push the boundaries of what is possible in filtration and purification technologies.

## 5 Future Perspectives

With the prevalence of work accomplished over the last two decades, the future is bright for functional graphenic materials. Early research in the 2010s set the stage for understanding and manipulating the chemistry,^2,181^ which progressed to the development of FGM biomaterials^91,182^ after seminal work showed the autodegradability of GO.^183^ This is not a closed chapter of research, however. Fully controlling and even characterizing the spatial distribution of functional groups on GO is an active area of research, as is synthesis of GO of precise size (flake size and number of layers). Further, researchers must continue to be vigilant to monitor degradation and toxicity as FGMs are developed and employed.

Our work on the “blanket effect” contributes to this ongoing research by demonstrating that GO sediments at high concentrations, smothering cells in culture systems without being inherently cytotoxic. This phenomenon highlights a critical limitation of traditional cell culture setups, underscoring the need for innovative approaches to better assess the biological interactions of FGMs. Similar effects have been observed with other nanoparticles, such as gold, where sedimentation at high concentrations alters cellular uptake.^184^ These findings emphasize the importance of understanding how nanoparticle behavior in experimental setups can influence observed outcomes, particularly for FGMs.

The perfect niche application for FGMs is still emerging. While they show promise across diverse fields, from tissue engineering to drug delivery, sustainability concerns and material optimization must remain at the forefront of development. With continued efforts to refine synthesis techniques, better characterize their properties, and address biological compatibility challenges, polymer-based FGMs have the potential to become the next “miracle material”.
